# Personalized machine learning guided intervention for optimizing lifestyle behaviors in depression: a pilot study

**DOI:** 10.1038/s44277-026-00062-3

**Published:** 2026-05-19

**Authors:** Jason Nan, Suzanna Purpura, Satish Jaiswal, Houtan Afshar, Vojislav Maric, James K. Manchanda, Charles T. Taylor, Dhakshin Ramanathan, Jyoti Mishra

**Affiliations:** 1 Neural Engineering and Translation Labs, University of California, San Diego, La Jolla, CA USA; 2 Department of Psychiatry, University of California, San Diego, La Jolla, CA USA; 3 Department of Mental Health, VA San Diego Medical Center, San Diego, CA USA; 4 Center of Excellence for Stress and Mental Health, VA San Diego Medical Center, San Diego, CA USA

**Keywords:** Predictive markers, Depression, Biomedical engineering

## Abstract

Personalized data-driven interventions for depression are much needed. Here, we leveraged N-of-1 machine learning (ML) to optimally target behavioral lifestyle interventions for depression. 50 individuals with mild-to-moderate depression enrolled in the single-arm, open-label Personalized Mood Augmentation (PerMA) pilot clinical trial (NCT05662254). Participants completed a two-week digital monitoring phase using smartphone-based ecological momentary assessments (EMAs, 4×/day) plus smartwatch tracking of mood and lifestyle factors (sleep/exercise/diet/social connection). Personalized ML models were generated from these data to identify lifestyle factors most predictive of individual mood, and results were translated to individualized mood augmentation plans (iMAPs) implemented by participants for six weeks with once-a-week health coach guidance. Intervention completers (n = 40) showed significant reduction in depression symptoms (primary outcome self-rated PHQ9 −3.5 ± 3.8, Cohen’s d = −0.89, CI [−1.25 −0.53], p < 0.001; clinician-rated HDRS −7.2 ± 6.8, d = −1.03, CI [−1.41 −0.65], p < 1E-6) with benefits sustained up to 12-week follow-up. Co-morbid anxiety was also significantly reduced (GAD7: d = −0.85, CI [−1.2, −0.49], p < 0.001) and quality of life improved (d = 0.68, CI [0.33, 1.02], p < 0.001). Additionally, objective cognitive measures impacted in depression including selective attention (d = 0.51, CI [0.18, 0.84], p < 0.001), interference processing (d = 0.53, CI [0.2, 0.85], p < 0.01) and working memory (d = 0.66, CI [0.31, 0.99], p < 0.001) showed significant enhancement. EMA tracking confirmed that improvement in depressed mood was specifically predicted by improvement in individually targeted lifestyles (β = 0.4 ± 0.09, p < 0.0005). Finally, decision algorithms and a large-language-model (LLM) could match human coach-led iMAP assignment with up to 95% accuracy. The PerMA trial presents a personalized lifestyle intervention approach for depression and merits scale-up and RCT testing to establish clinical efficacy. PERMA was registered with ClinicalTrials.gov under registry number NCT05662254.

## Lay summary

This pilot study introduces a personalized, data-driven approach to treating depression. We use smartwatches and brief daily surveys to track a participant's lifestyle, and leverage machine learning to identify the domain (sleep, exercise, diet, social) that impacts their mood most. Instead of a "one-size-fits-all" therapy, this method allows patients to focus on a specific lifestyle aspect that will have the biggest impact on their mental health. Our results show that this targeted approach successfully reduces symptoms of depression.

## Introduction

Depression carries the largest burden of mental health disorders with prevalence of depressive episodes observed in 18% of the total US population and 21% of young adults [1, 2]. Besides the negative health impacts of depression, its socio-economic cost burden was recently estimated at greater than $380 billion with healthcare as the major cost driver alongside household and work-related costs [3]. Notably, the majority (~67%) of all depression cases fall in the category of mild-to-moderate depression [3] for which integrative behavioral health treatments are recommended first-line as effective and scalable solutions [4]. These include physical activity/exercise, dietary modification, adequate sleep and social interaction, as well as mindfulness-based meditation, all of which have shown treatment efficacy in separate clinical trials of depression [4–16]. Meta-analyses of lifestyle intervention trials find low-to-medium effect sizes for depression alleviation (Cohen’s d ~ 0.3–0.5). Yet, a major limitation of these homogenously assigned (non-personalized) behavioral intervention studies is the heterogeneity across individuals of the specific lifestyle factors most closely linked to their depression. Consequently, a single lifestyle treatment domain is unlikely to be universally beneficial, and these studies fail to account for individual differences that may favor one behavioral health solution over another [17–19]. This limitation has sparked a new push towards developing personalized treatments that are tailored to each individual. Indeed, personalized behavioral health solutions for mild-to-moderate depression could prove to be more effective, scalable and accessible, hence, optimizing such treatments offers incredible benefit for society.

Studies across multiple fields of healthcare have shown that personalizing treatment leads to higher treatment adherence as well as improved satisfaction [20–23]. A depression intervention study that used a personalized advantage index (PAI) to assign either cognitive or interpersonal therapy to individuals showed decreased long-term symptom severity in patients receiving PAI-indicated vs. PAI-non-indicated treatment [24]. A recent systematic review and meta-analysis of multiple personalized psychological interventions found that personalization significantly improves the effect size of treatment compared to standard therapy [25]. Yet, of note, all of these personalized intervention studies use predictive models based on multi-subject data. While this may be adequate to distinguish between subtypes of depression, such models do not capture individual lifestyle attributes that drive day-to-day fluctuations in depressed mood. Further, such models based on data from specific populations may not be generalizable and thus may fail to properly assign the optimal treatment [26]. An alternate approach to population data-based machine learning is to design models that link lifestyle features with depressed mood based on an individual’s own data over time – also referred to as idiographic or N-of-1 modeling. Here, based on our prior research developing an N-of-1 machine learning (ML) pipeline for depressed mood [17], we aimed to assign behavioral interventions exclusively based on individual data acquired over time and thereby, steer clear of issues related to between-subject heterogeneity and generalizability.

Overall, this pilot study aimed to deliver personalized behavioral intervention based on data-driven N-of-1 ML models tailored to individual lifestyles. We have previously demonstrated high accuracy ML modeling of individual depressed mood based on ecological momentary assessments (EMA) and passive smart watch data [17, 18, 27]. Furthermore, leveraging model explainers such as Shapley plots, we have identified the top predictive intervention targets for each individual [17, 18]. Trained health coaches can then utilize this predictive information to determine the optimal behavioral intervention for each individual. Delivering behavioral health interventions through health coaches has the added benefit of being cost-effective compared to repeat visits to licensed mental healthcare providers, i.e., psychiatrists or clinical psychologists, and can be particularly beneficial in low-income settings [28–30]. Additionally, we used a multidimensional approach to evaluate trial outcomes; symptom assessments were complemented with evaluation of change in subjective mental health behaviors and quality of life, as well as change in objective measures of neuro-cognition that are impacted in depression [31–37]. Overall, to the best of our knowledge, this is a first study to implement a N-of-1 ML-personalized behavior intervention for depressed mood using digital lifestyle markers. If shown to be effective, such a personalized approach can be a promising way to remotely deliver scalable treatment for depression that is optimized to individual lifestyles.

## Methods

### Participants

A total of 50 individuals participated in this pilot study (mean age: 43.3 ± 16.1, range: 23–76, 12 males). Participants were recruited from the San Diego community using the ResearchMatch registry and social media, email and print flyers. All participants were fluent in English and study inclusion was based on current mild-to-moderate depression symptoms per self-reported Patient Health Questionnaire (PHQ9) scores in the 5–17 range [38] further confirmed via structured clinical interview for DSM V Axis I disorders (SCID) [39]. Exclusion criteria evaluated via SCID consisted of evidence for active substance use disorder, psychotic disorder, bipolar disorder, eating disorder, or displaying acutely suicidal behaviors. For participants 60 years of age and older, we additionally conducted the Montreal Cognitive Assessment (MoCA) and confirmed normal scores ≥26 [40]. All participants provided written informed consent in accordance with the Declaration of Helsinki before participating in the study. All experimental procedures were approved by the Institutional Review Board of the University of California San Diego (UCSD) (protocol #180140). Data collection took place during Winter 2022-Fall 2024.

### Sample size and power

The sample size of 50 participants accounted for up to 20% attrition, so as to obtain at least 40 complete study samples that were powered to detect a statistically meaningful medium effect size (Cohen’s d > 0.45) primary outcome difference in pre vs post comparisons of PHQ9 scores at 0.8 power and alpha level of 0.05, computed a priori [41].

### Study procedure

We pre-registered the single-arm study in ClinicalTrials.gov as the Personalized Mood Augmentation (PerMA) trial: NCT05662254 [42].

During the first phase, participants downloaded the HIPAA compliant Unity-based BrainE© app on their iOS/Android smartphone [43]. Participant data stored is deidentified and labeled with an alpha numeric participant ID corresponding to their study ID. All data is stored on a secure Amazon Web Server (AWS) account. Within the app, participants accessed daily EMAs on a module called MindLog on which they provided mood and lifestyle ratings up to 4 times per day until they completed 60 sessions (2–4 weeks). The app sent regular notifications daily to all participants following the methodology of recent research on longitudinal mood monitoring [17, 18, 44]. Notification times were fixed for participant convenience but there was no expectation to respond at these times. We opted for 60-session models to minimize participant burden, as prior comparisons with 120-session models showed no significant gain in accuracy (MAPE difference = 1.5%, p = 0.3) [17]. Participants also wore a Samsung Galaxy wristwatch throughout the study. Consumer wearables were chosen due to their accessibility and low-profile nature compared to medical grade devices. At completion of 60 sessions of mood and lifestyle tracking, individual mood augmentation plans (iMAPs) were generated using N-of-1 machine learning models [17, 18]. The models were developed in accordance using best practices for machine learning (cross validation, hyperparameter tuning, data imputation and standardizing) and built upon the validated frameworks detailed in our previous work, ensuring full technical transparency and reproducibility [17, 18]. A flowchart can be seen in Supplementary Fig. 2. These models were built targeting one of four major lifestyle domains: sleep, exercise, diet, and social connection.

**Fig. 1:**
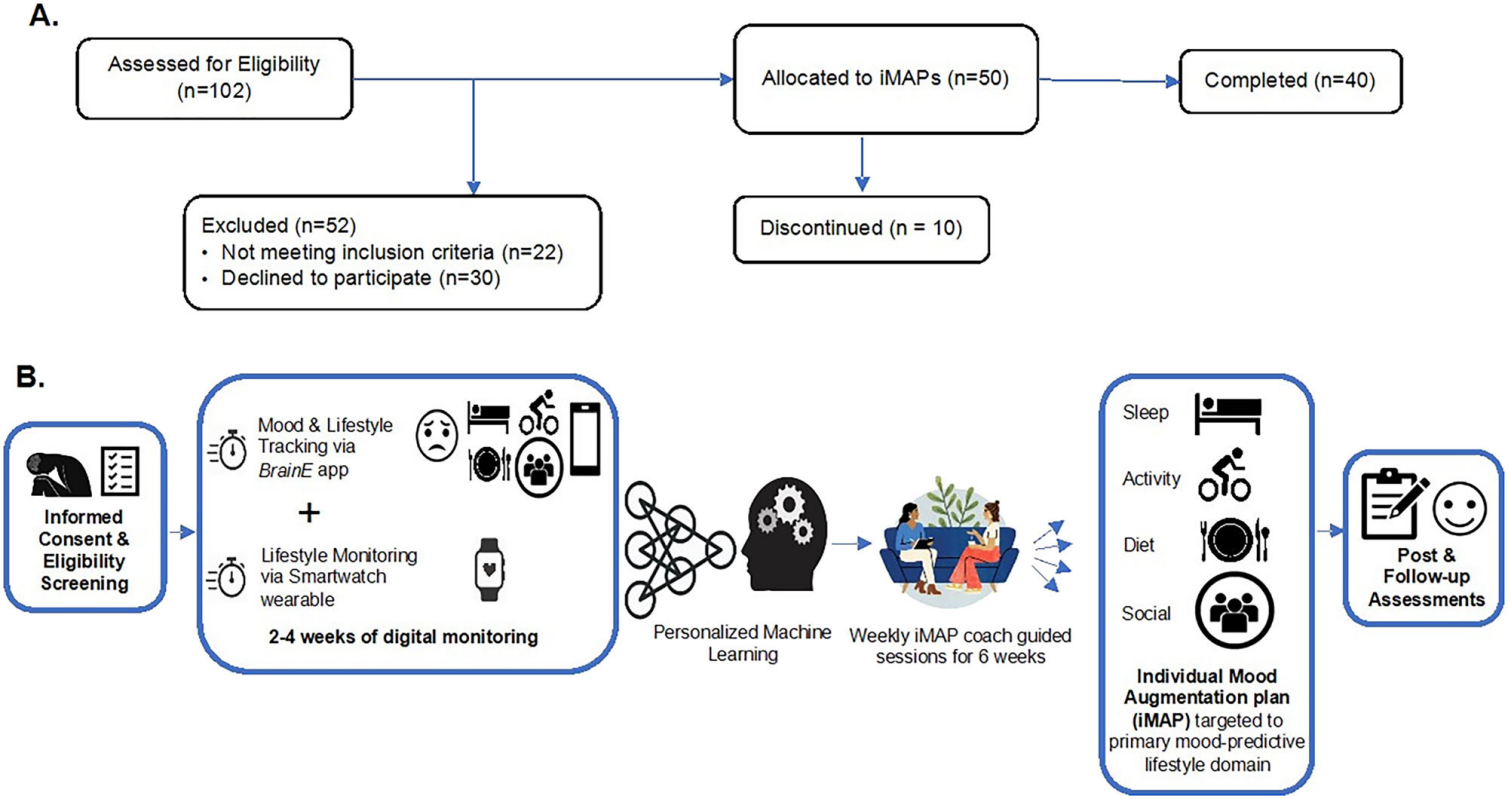
Personalized Mood Augmentation (PerMA) trial flow. **A** Participant flow is shown from eligibility assessments to completion. **B** Study design is shown from left to right. Informed consent and eligibility screening was followed by a 2–4 week digital monitoring phase with both smartphone and smartwatch tracking until 60 EMA sessions were completed followed by personalized machine learning model generation, which was then followed by the second phase of individual mood augmentation plan (iMAP) assignment and weekly guided sessions with a health coach for 6 weeks, and subsequent post-intervention assessments and then follow-up assessments at 6 and 12 weeks after end of intervention.

The second phase of the study lasted 6 weeks where participants had once-a-week iMAP guided sessions (GS) with a trained health coach on one-on-one virtual video calls of ~20 min duration each. The GS focused on individualized features generated from the N-of-1 model in Phase 1 within the targeted domain. Health coaches in this study were two medical student trainees (co-authors HA, VM) who received basic training on how to read and interpret iMAPs in layman terms, and then how to motivate and guide individuals through standard, evidence-based behavioral lifestyle interventions focused on sleep, exercise, diet or social connection – protocols described in detail below. The coaches received 8 h of training consisting of both didactic training as well as practical skills development with role play of realistic participant-coach interactions supervised with feedback from the study psychiatrist (co-author DR). The study psychiatrist also monitored the first few video GS performed by each of the newly trained health coaches to ensure coaching consistency and provide any additional coaching feedback, if necessary. During the 6-weeks iMAP implementation phase, participants continued to complete once-a-day mood and lifestyle EMAs to track daily progress. Figure 1 visualizes the study flow and protocol design.

### Clinical and behavioral outcomes

The primary clinical outcome was change in self-reported depression ratings on the PHQ9 scale [38] from pre- to post-intervention. The PHQ9 scale was completed by self-report at eligibility screening; before start of study digital monitoring procedures (pre); at end of digital monitoring/beginning of the second phase at the first coach guided session (GS1); every two weeks during iMAP intervention (at GS3, GS5); at post-intervention (post); and also at 6-week and 12-weeks follow-up after end of intervention.

Secondary clinical and behavioral outcomes included the Generalized Anxiety Disorder 7-item scale (GAD7 [45]) acquired at the same time-points as the PHQ9 above, given that anxiety and depression are highly co-morbid [46, 47]. In addition, at pre- and post-intervention time-points, outcome scores were acquired for clinician-rated depression on the Hamilton Depression Rating Scale (HDRS [48]), self-rated quality of life reported on the Short Form 12 Mental Component Summary (MCS12 [49]), and mindfulness self-rated per the Mindful Attention Awareness Scale (MAAS [50]). Mindfulness was intentionally assessed as it can facilitate behavior change through greater self-awareness and control over decision-making [51, 52]. Suicidality was also monitored at the same time-points as the PHQ9 and GAD7 scales using the Columbia Suicide Severity Rating Scale (CSSRS [53]), not as an outcome but as a safety measure to monitor any worsening of suicidal risk at any point during the study.

All clinical and behavioral assessments were completed in a de-identified manner with alphanumeric study IDs using the secure web-based Ready Electronic Data Capture (REDCap) system.

### Cognitive outcomes

Secondary outcomes also included assessments of neuro-cognition completed using the BrainE app, specifically tests of selective attention, interference processing, working memory and emotion bias, described in several prior publications [17, 54–66] and also in Supplementary Methods section S.1 and Supplementary Fig. 1.

### Intervention phase 1 - digital monitoring

During this phase, participants wore a smartwatch and completed EMAs of 1–2 min duration on the BrainE© mobile app’s MindLog module [17, 18] up to 4X per day for max 60 EMA sessions completed over 2–4 weeks. Participants received daily app notifications sent at 8am, 12pm, 4pm and 8pm until all EMAs were completed. Notifications were for participant convenience, and they could complete EMAs at any time with the constraint that the app did not allow a new EMA within 2 h after a prior entry. EMAs probed mood and lifestyle variables pertaining to sleep, diet, exercise, and social connection detailed in the Supplementary Methods section S.2. and breath related exercises in S.3. Smartwatch data used included heart rate, step attributes (count, speed, distance and calories burned).

#### iMAP modeling - data ingestion, feature extraction, personalized ML pipeline

Data from both smartphone and smartwatch sources were carefully aligned. For this all independent data variables were either aggregated or extrapolated based on their sampling frequencies to match the sampling frequency of the dependent variable (DV), i.e., depressed mood EMA ratings. All features extracted from the EMA and smartwatch data are enumerated in Supplementary Methods section S.4; the ML pipeline was adopted from our previous research [17, 18] and is further detailed in this Supplementary Methods section.

**Fig. 2:**
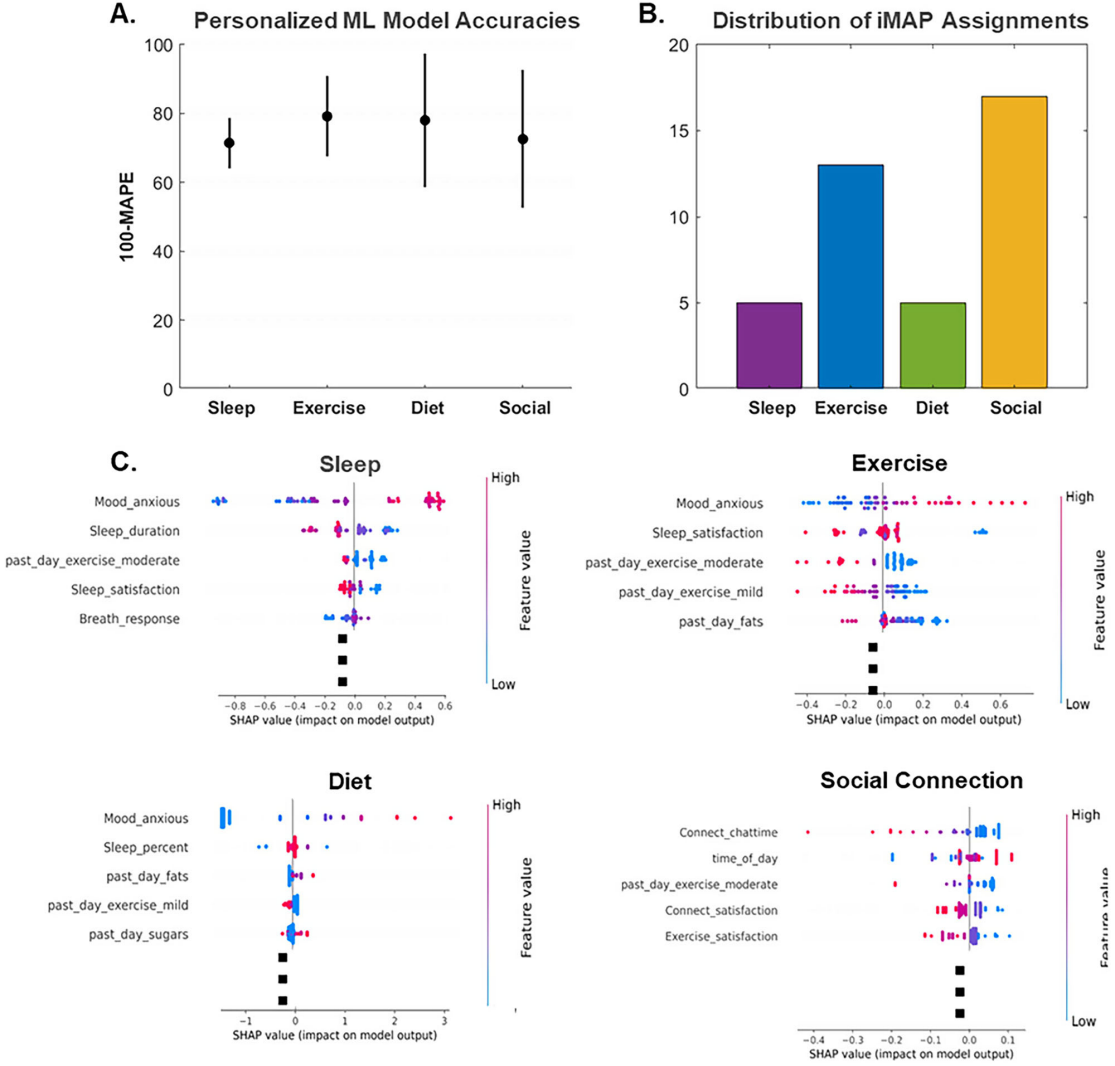
Results of personalized ML modeling. **A** ML Model accuracies split by the 4 assigned iMAP domains calculated as (100 – mean ± sd of best-fit model mean absolute percentage error, MAPE). ML model accuracies were not significantly different between domains as confirmed by one-way ANOVA (p > 0.66). **B** Distribution of iMAPs assigned to the 40 study completers: sleep (5), exercise (13), diet (5), and social connection (17). **C** Four example Shapley plots showing top 5 predictive features of depressed mood from four distinct participants that were recommended either sleep, exercise, diet or social connection based iMAPs. Shapley dot plots of all ranked feature predictors show directionality of prediction; each dot represents a single datapoint, red dots indicate larger positive feature values while blue dots indicate larger negative feature values. X axis is the Shapley values with center point at 0. Positive Shapley values increase the model output (higher depression), negative Shapley values decrease the model output (lower depression). Each plots has multiple iMAP domain-specific variables appear high in the rankings, and directionality aligned with the goals of the behavioral intervention (i.e., better sleep, more exercise, lower fats and sugars, and more social connection are linked to lower depression).

For each person’s best-fit personalized ML model we utilized Shapley statistics, specifically SHapley Additive exPlanations (SHAP), [67]. Shapley values indicate the relative importance and directionality of each feature as it predicts the depressed mood DV, notably reflecting associations but not causal relationships or inherent modifiability of the underlying variables [17, 18]. Features with large absolute Shapley values are essential for the predictive model, hence, we rank-sorted these feature values and the top 10 (that accounted for majority of the variance attributed to the DV by the model) were inspected by the coaches to determine iMAPs. Examples of Shapley plots that map to the 4 iMAP domains are shown in Fig. 2. The personalized ML model results, thereby, revealed the specific lifestyle attributes that are most impactful in predicting each individual’s depressed mood, and the top-ranking predictors could then be used to guide each participant’s iMAP.

### Intervention phase 2 – coach guided iMAPs

The trained behavioral health coaches reviewed the Shapley results to identify the top iMAP target domain, i.e., sleep/ exercise/ diet/ social connection, for each person. In case features from more than one domain were present within the top 10 predictors of depressed mood, coaches also based their decision on number of domain features present as top predictors, whether these predictors independently showed high correlations with depressed mood, whether participants reported low satisfaction with the chosen lifestyle domain, and whether it was feasible for participants to initiate intervention in the chosen domain (e.g., fixed sleep schedules due to work may constrain a person’s ability from engaging in a sleep-focused plan, in which case the second most mood-predictive domain would be the focus of intervention). Description of each 6-week iMAP in either sleep/ exercise/ diet/ social connection domain is provided in Supplementary Methods section S.5 and is grounded in evidence-based intervention protocols in these lifestyle domains in the context of depression [5, 6, 8, 11, 16, 68–70]. Each participant met once-a-week with their coach for a ~ 20 min GS video call to discuss progress and any impediments to behavior change. Participants also completed once-a-day EMAs (same as those in phase 1) over the 6 weeks to track progress.

### Data analyses

For all analyses, we report effect sizes as Cohen’s d for post (or follow-up) vs. pre-intervention comparisons with 95% confidence intervals (CI). For regression analyses, standardized regression coefficients are reported where applicable: >0.1 as small, >0.3 as medium, and >0.5 as large effect size [71].

#### Clinical and behavioral outcomes analysis

Depression (PHQ9, primary outcome) and anxiety (GAD7, secondary outcome) were tracked at multiple time points - screening (PHQ9 only), pre-intervention, end of intervention phase 1/GS1, GS3, GS5, post, 6-week and 12-week follow-ups. One participant was missing screening data, 2 were missing GS3, and 8 were missing GS5 timepoints for PHQ9, resulting in 4.6% missing. One participant was missing pre data, 2 were missing GS3, and 7 were missing GS5 for GAD7, resulting in 5% missing. Given the limited number of missing data points and their sequential nature, imputation was done independently for each participant with linear interpolation. Friedman’s repeated measures test was used to investigate significant change from screening to post-intervention. Sphericity was checked with Mauchly’s test and p values were corrected per the Greenhouse-Geisser method. We also checked whether iMAP intervention domain (sleep/exercise/diet/social) was a significant covariate in the repeated measures model. In post-hoc testing, we tested each time point against the ‘Pre’ time point for significance using the non-parametric signed rank test.

Follow-ups at 6 and 12 week had missing data at 30 and 45% for both PHQ9 and GAD7. As such, they were not included in the above analysis nor imputed. Instead, these time points were assessed with the signed rank test against the pre-intervention time point without imputation.

For the additional pre vs. post-intervention secondary outcomes, i.e., clinician-rated depression (HDRS), quality of life (MCS12), and mindfulness (MAAS), normality of each variable was verified with the Anderson Darling test; non-normal variables were tested for post vs. pre differences using the signed rank test, else a paired t-test was used. Additionally, ANOVA was used to verify if iMAP intervention domain was a significant covariate of post vs. pre change for these outcomes.

#### Cognitive outcomes analysis

For all secondary outcomes of cognition, i.e., selective attention, interference processing, working memory and emotion bias, task efficiency as the product of accuracy and processing speed was evaluated as the main outcome measure.

#### Relationship between primary and secondary outcomes

A robust linear regression model was used to investigate the relationship between the post vs. pre-intervention change in the primary PHQ9 outcome and all secondary outcomes, controlling for demographics of age, gender and ethnicity. Robust regression was used to minimize any outlier influence [72]. PHQ9 change from pre-intervention to follow-ups was also similarly investigated while controlling for demographics.

#### Baseline (pre-intervention) measures associated with remission

We assessed whether any demographics (age, gender, ethnicity) or secondary behavioral (GAD7, HDRS, MCS12, MAAS) or cognitive outcomes at baseline (i.e., at pre-intervention) related to remission, i.e., post-intervention PHQ9 score <5 reflecting healthy, non-depressed status. For this, we used one-way ANOVA with remitted vs. non-remitted participants as the between-subjects factor for all continuous variables, except categorical variables of gender and ethnicity that were tested using $\chi$^2^ test. All p-values were fdr-corrected for multiple comparisons across demographic and baseline outcomes. We further verified that measures were still significant when accounting for baseline depression symptoms in a generalized linear regression model.

#### EMA analysis (during Intervention Phase 2)

This analysis aimed to investigate whether targeted change in the participant’s iMAP lifestyle domain versus non-specific general lifestyle change was associated with improvement in depressed mood in Intervention Phase 2. A linear fit was applied to each participant’s target and off-target domain’s representative data to calculate the slope of change over the intervention period. A robust linear regression model was fit to investigate whether slope of change of depressed mood was predicted by the change slopes for the target and off-target metrics across all participants. Data were normalized across subjects for this linear regression to report standardized betas reflecting effect size. Details about how the representative data was calculated can be found in the Supplementary Methods S.6.

### Automated iMAP lifestyle domain assignment

As personalized ML Shapley results rank lifestyle features such that the exact domain of intervention is subject to interpretation by human coaches, we also explored whether a decision-based algorithmic approach or a Large Language Model (LLM) through Google’s Gemini 2.5 flash model [73] can further automate iMAP lifestyle domain assignment from the Shapley data. Here, we considered the human coach assignment as the benchmark and compared the LLM/decision algorithm results to that of the human coach; notably, this automation exercise was performed after all participants had already completed the intervention study, so that an automated process for optimal iMAP domain selection could be deployed in future larger scale work. Details of the decision algorithm and the Gemini LLM prompt are provided in Supplementary Methods section S.7. The decision algorithm approach was meant to model the rationale for intervention assignment, and a table of iMAP domain ranking values for each participant is also included in Supplementary Table 1.

## Results

Demographic data for all 50 participants are tabulated in Table 1. Average age of participants was 43 ± 16 years and per known greater prevalence of depression in females, majority of study participants were female. Ethnicity data matched percentages of ethnic populations in the US San Diego county where the study was conducted. Average baseline depression and anxiety scores were in the mild-to-moderate range and were significantly correlated (rho = 0.61, p < 0.0001).

**Table 1:** Demographics for n = 50 trial participants. sd: standard deviation.

Demographics	
Age (years, mean ± sd)	43.3 ± 16.1
Gender n (%)	
Male	12 (24%)
Female	38 (76%)
Ethnicity n (%)	
Caucasian	32 (64%)
Black/African American	5 (10%)
Asian	6 (12%)
American Indian/Alaska Native	1 (2%)
More than one race	4 (8%)
Unknown	2 (4%)
Pre-intervention Mental Health (mean ± sd)	
PHQ9	9.2 ± 5.1
GAD7	7.1 ± 5.3

### Model accuracy, intervention feasibility, and adherence

All 50 enrolled participants completed pre and post assessments and 46 completed the digital monitoring i.e., 100% of EMAs during phase 1 of intervention; the remainder 4 individuals completed 31% of EMAs. Across all participants, the best fit personalized ML model showed high accuracy of 75.3 ± 15.2% (100 – mean ± sd of best-fit model mean absolute percentage error, MAPE). ML model mean and sd accuracies split by assigned intervention domain as well as distribution of assigned iMAP domains are shown in Fig. 2; example Shapley plots of four participants assigned to one of each iMAP domain are also shown. 80% participants (n = 40 of 50) also completed all 6 coach guided sessions, i.e., phase 2 of intervention with 19.9 ± 13 of 30 (mean ± sd) EMA reported over the 6 weeks of phase 2. 10 participants did not complete phase 2 due to time constraints; of these, four participants did not engage with any phase 2 intervention, and six participants partially engaged with phase 2 intervention (i.e., 0 GS (n = 4), 1 GS (n = 4), 2 GS (n = 1), or 3 GS (n = 1)). There was no significant difference in baseline demographics or depression scores between trial completers vs. non-completers (p > 0.8).

### Improvements in primary depression outcome

**Fig. 3:**
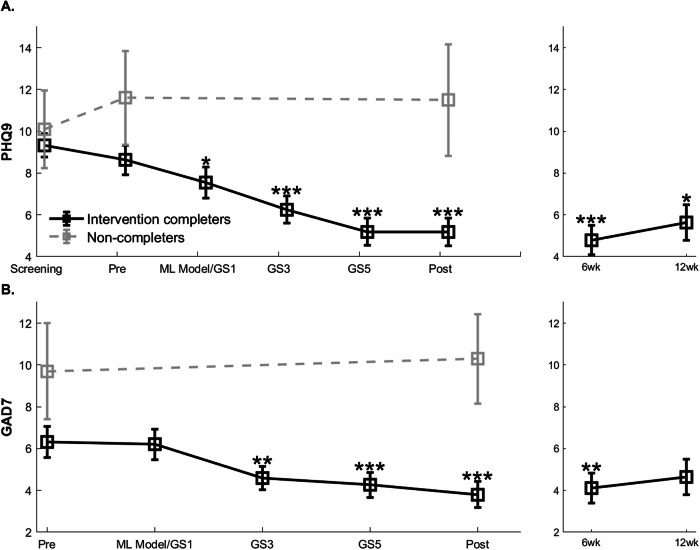
Depression (PHQ9) and anxiety (GAD7) outcomes throughout the study period. Mean ± sem scores are plotted in black for trial completers (n = 40) and as dashed grey lines for non-completers (n = 10). **A** PHQ9 scores are shown across the study period from screening to post-intervention with follow-up scores shown at right at 6 weeks (n = 28) and 12 weeks (n = 22). **B** GAD7 scores are shown across the study period from pre to post-intervention with follow-ups at right. GS: guided session with GS1 corresponding to the first coach meeting to discuss the ML model results and iMAP with each participant. Stars indicate fdr-corrected signed rank significance across all time points relative to pre-intervention: *: p < 0.05; **p < 0.01; ***p < 0.001.

Figure 3A shows the progression of self-reported depression symptom scores (PHQ9) across all 40 trial completers as well as separately for the 10 non-completers. Intent to treat effect size for PHQ9 scores including all 50 participants was d = −0.67, CI [=−0.97, −0.36].

We conducted detailed analyses in trial completers. The Friedman’s repeated measures ANOVA showed a significant decline in depression from pre to post-intervention (mean ± sd change = −3.5 ± 3.8, *χ*^2^ (df = 5) = 72.6, p < 1E-13, effect size d = −0.89, CI [−1.25, −0.52]), with post-hoc testing showing significant decline from pre-intervention at all GS 1, 3, 5 and post-intervention time points. While PHQ9 scores significantly improved even after phase 1, i.e., at end of digital monitoring corresponding to GS1 when the coach revealed the iMAP to each participant, end of phase 2 results were significantly better than end of phase 1 results (phase 2 vs. phase 1 PHQ9 change relative to baseline, signed rank z-value = 3.9, p = 1E-4). Overall, 22 of 40 (55%) participants showed symptom remission (PHQ9 score<5) at post-intervention. Notably, PHQ9 symptom improvement was sustained at 6-week (d = −1.06, CI [−1.51, −0.6], p < 1E-5, n = 28) and 12-week follow-ups (d = −0.52, CI [−0.95, −0.09], p < 0.05, n = 22).

Additionally, there was no effect of iMAP domain covariate (sleep/exercise/diet/social connection) in the pre- to post-intervention repeated measures ANOVA (p = 0.78) and demographics of age, gender, ethnicity also did not influence these outcomes (p > 0.27). Supplementary Fig. 3 shows the similar progression of PHQ9 scores for trial completers split by the 4 iMAP target lifestyle domains. Notably, the 10 study non-completers did not show significant change in PHQ9 scores at post- vs. pre-intervention (d = −0.01, CI [−0.85, 0.83], p > 0.8) so there was no spontaneous remission of symptoms.

### Improvements in secondary outcomes

Figure 3B shows the progression of self-reported anxiety symptoms (GAD7) for trial completers (n = 40) and separately for the non-completers (n = 10). Similar to change in depression scores, the Friedman’s repeated measures ANOVA showed a significant decline in anxiety from pre to post-intervention (mean ± sd change = −2.5 ± 3, *χ*^2^ (df = 4) = 31.9; p < 1E-5, d = −0.85, CI [−1.21, −0.49]), with post-hoc testing showing significant decline from pre-intervention at GS 3, 5 and post-intervention time points, which was sustained at 6-week (d = −0.59, CI [−0.98, −0.19], p < 0.005, n = 28) but not 12-week follow-up (d = −0.3, CI [−0.71, 0.12], p = 0.16, n = 22). There was no effect of iMAP domain covariate in the repeated measures ANOVA for anxiety (p = 0.19) and the 10 trial non-completers did not show significant change in GAD7 scores at post- relative to pre-intervention (d = 0.08, CI [−0.76 0.9], p > 0.7).

**Fig. 4:**
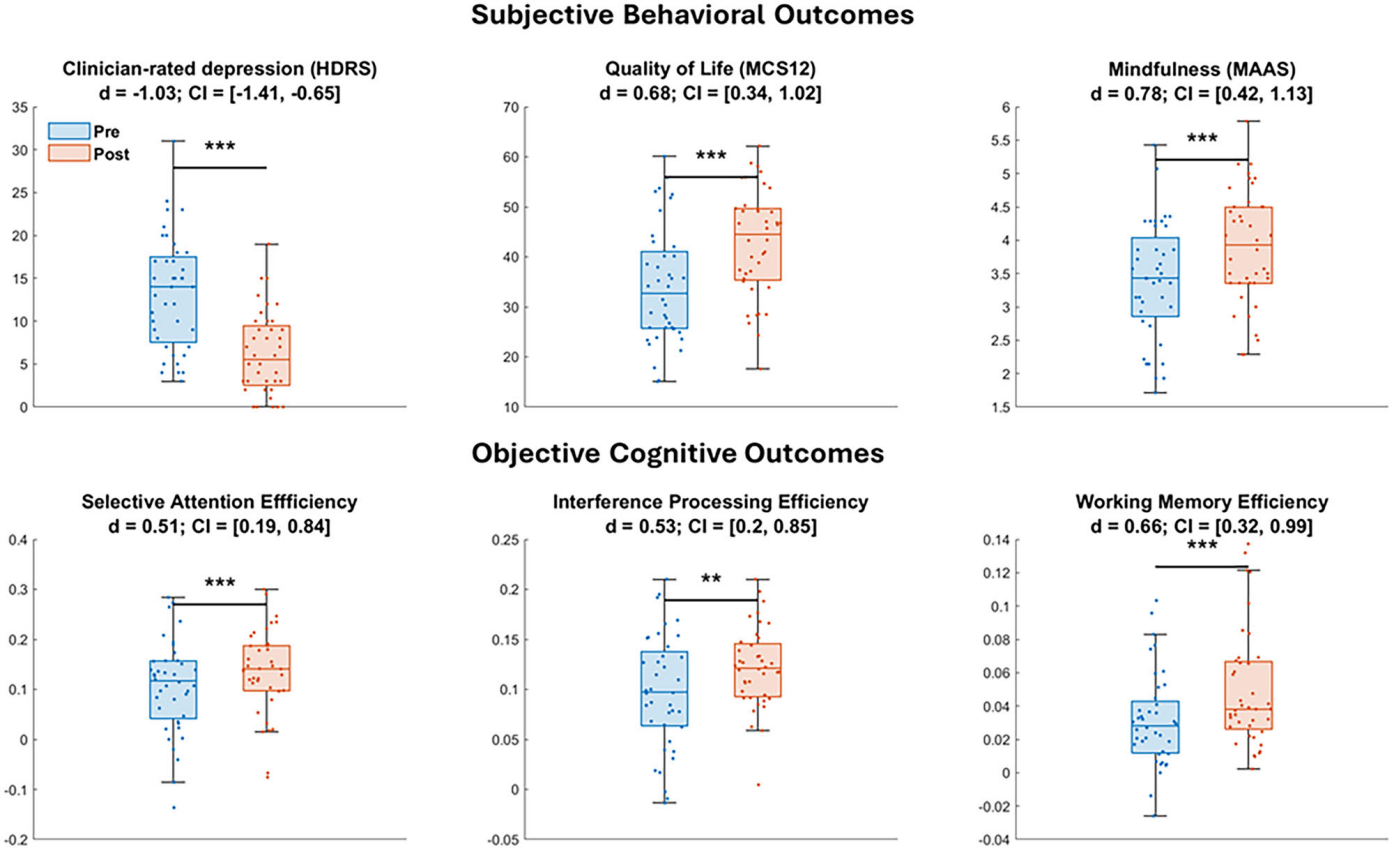
Pre- vs. post-intervention results for secondary outcomes of subjective behavior (top row) and objective cognition (bottom row) in trial completers (n = 40) shown as box and swarm plots. Box plots show median with lower and upper quartiles as the box edges, whiskers denote the data range (excluding outliers) and the scatter points show individual participant values. Effect sizes with lower and upper 95% confidence intervals in brackets are shown under each measure label. Stars indicate fdr corrected significance. *: p < 0.05; **p < 0.01; ***p < 0.001.

Further, significant improvement was observed for secondary outcomes measured at post relative to pre-intervention in trial completers (n = 40, Fig. 4) for subjective measures of clinician-rated depression (HDRS: d = −1.03, CI [−1.41, −0.65], p < 1E-6), self-rated quality of life (MCS12: d = 0.68, CI [0.34, 1.02], p < 0.001) and mindfulness (MAAS: d = 0.78, CI [0.42, 1.13], p < 0.0001). In addition, objective measures of cognition showed significant improvement in performance efficiency for selective attention (d = 0.51, CI [0.19, 0.84], p < 0.001), interference processing (d = 0.53, CI [0.2, 0.85], p < 0.01) and working memory (d = 0.66, CI [0.32, 0.99], p < 0.001), but not emotion bias (p = 0.3). All significant results were fdr-corrected for multiple comparisons, and there was no effect of iMAP lifestyle domain covariate or participant demographics on any of these measures (p > 0.16).

### Relationship between primary and secondary outcomes

**Fig. 5:**
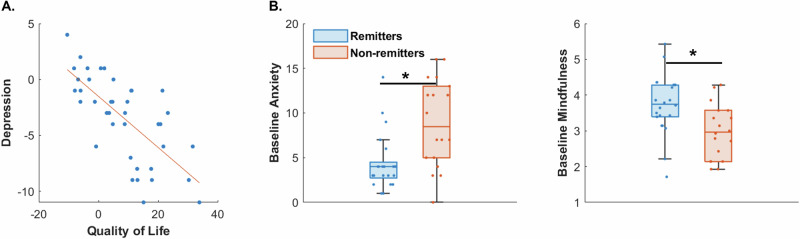
Factors supporting depressive symptom improvement. **A** Change in post vs. pre PHQ9 depression symptoms were associated with improvement in quality of life; scatter plot are shown with best fit line. **B** Baseline, i.e., pre-intervention measures associated with remission included anxiety (GAD7, left) and mindfulness (MAAS, right). Box plots show median with lower and upper quartiles as the box edges, whiskers denote the data range (excluding outliers) and the scatter points show individual participant values. Remitters depicted in blue and non-remitters in red. *p < 0.05 fdr-corrected.

We investigated the relationship between post vs. pre-intervention change in the primary PHQ9 outcome and all secondary outcomes controlling for demographics using robust linear regression. This overall model was significant (F = 3.5, p = 0.003) and the only variable that showed a significant effect was quality of life (MCS12: β = −0.59 ± 0.14, p = 0.0004), i.e., improved quality of life was related to lower depression symptoms at post-intervention (Fig. 5A). Further, robust regression models at 6 and 12-week follow-ups were also significant, i.e., post vs. pre change in quality of life also predicted sustained improvement in PHQ9 scores from baseline to 6-week (β = −0.71 ± 0.15, p = 0.0002) and 12-week (β = −0.59 ± 0.15, p = 0.002) follow-ups.

### Measures associated with remission

We used ANOVAs to investigate whether any pre-intervention data, i.e. demographics or secondary behavioral or cognitive outcomes showed separation between remitted vs. non-remitted trial participants (post-PHQ9 score<5). This analysis revealed that mindfulness (MAAS: F(df = 1) = 9.04; p = 0.04) and anxiety (GAD7: F(df = 1) = 11.5; p = 0.03) at baseline were significantly different between remitters (22/40) vs. non-remitters (18/40), with remitters showing lower anxiety and greater mindfulness at baseline (Fig. 5B). Of note, baseline anxiety and mindfulness were still significant predictors of remission (p < 0.05) if baseline depression was accounted for in a generalized linear regression model. A similar analysis for intervention responders (≥50% symptom reduction, 16/40) vs. non-responders (24/40) showed no significant relationship after fdr-correction.

### Relationship between change in depressed mood and iMAP targeted lifestyle domain

**Fig. 6:**
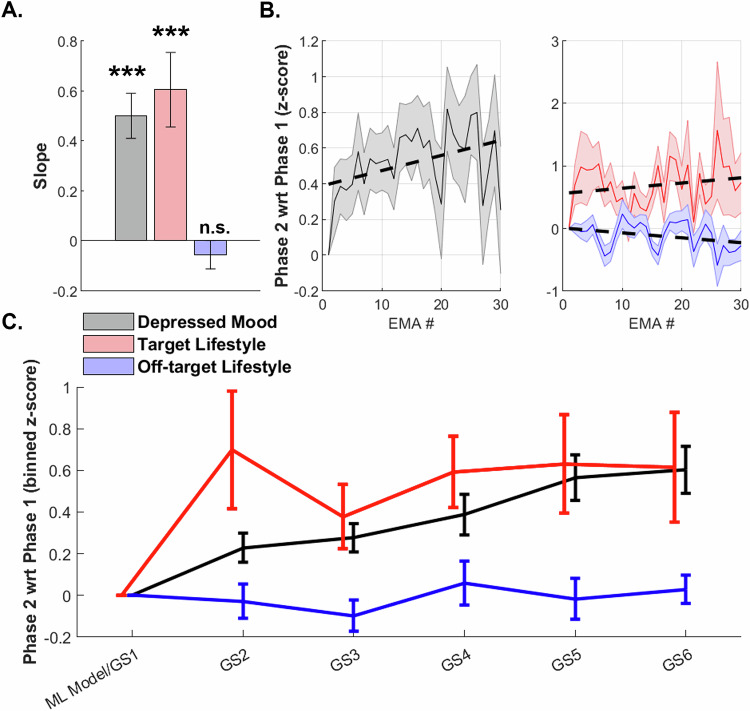
EMA metrics during intervention phase 2. **A** Average slope, i.e., z-scored change in phase 2 relative to phase 1 in depressed mood (grey), target (red), and off-target (blue) lifestyle domains. *** indicates p < 0.001; n.s.: non-significant. **B** Evolution of z-scored depressed mood (grey), target (red) and off-target (blue) EMAs across all participants, shaded regions reflect sem. wrt: with respect to **C** Z-scored change in phase 2 relative to phase 1 EMA metrics binned by guided sessions (GS); mean ± sem bars are shown for depressed mood (black), target (red) and off-target (blue) EMAs.

Figure 6 shows that iMAP-related alleviation of depressed mood (d = 0.85, CI [0.49 1.2], p < 0.0001) was accompanied with improvement in the individually targeted lifestyle domain (d = 0.62, CI [0.28 0.95], p < 0.0005) but no significant change in the off-target domains (p > 0.3). These data were z-scored EMAs corresponding to coach GS supported intervention phase 2 relative to phase 1 digital monitoring. The evolution of these EMA metrics across phase 2 is shown in Fig. 6B. A robust linear regression model assessing the overall relationship between the slope of depressed mood and the slopes of target and off-target lifestyle domain variables across all subjects was significant (F = 19.8, p < 1E-5) with a significant regression coefficient observed only for the primary target domain slope (β = 0.4 ± 0.09, p < 0.0005) but not for the off-target slope (p > 0.1). Interestingly, this relationship between depressed mood improvement and change in target lifestyle emerged as early as the first 10 EMAs in phase 2 (β = 0.35 ± 0.14, p = 0.02) and persisted thereafter. For visualization purposes only, the evolution of EMA data binned by GS is also shown (Fig. 6C). To note, while these EMA-based depressed mood and target lifestyle data related to each other and provided confirmation of iMAP intervention specificity, we did not find a relationship between these metrics and the primary and secondary clinical and cognitive outcomes detailed above.

### Automated iMAP assignment

Here, we investigated whether a decision algorithm (DA) or an LLM (Google Gemini) could be used for iMAP target lifestyle domain assignment that resembled the selection made by the human coach. The naïve DA model resembled the human coach-based target domain assignment in 87.5% of cases (35 of 40 participants). It assigned a numeric value for each domain based on attributes human coaches considered from the data (see naïve DA equation 1 in Supplementary Methods S.7).

The LLM approach (prompt detailed in Supplementary Methods S.7) yielded 92.5% match with the human coach iMAP domain assignment. Finally, we also fine-tuned the DA model by empirically iterating the weights of the naïve DA equation based on the actual target domain preferences made by the human coaches across all study participants (see fine-tuned DA equation 2 in Supplementary Methods S.7). The fine-tuned DA equation achieved 95% match with the human coach-based lifestyle domain assignment.

## Discussion

This study introduced the Personalized Mood Augmentation (PerMA) pilot trial as a novel, data-driven N-of-1 ML based behavioral lifestyle intervention approach for mild-to-moderate depression. The trial included a two-week digital monitoring phase acquiring smartphone EMA and smartwatch data, based on which personalized ML was executed and Shapley statistics were applied to reveal lifestyle features rankings that best predict individual mood fluctuations over time. Based on top personalized ML Shapley features, iMAPs were assigned to each participant for a six-week intervention with once-a-week human coach guidance. While artificial intelligence (AI) chatbot based therapy is gaining traction [74–76], the depth of understanding and empathy provided by a human coach are still unmatched by chatbots [77–79], hence, we decided to integrate human coaches that had remote weekly video check-ins with each patient.

Overall this pilot study showed high feasibility with high accuracy best-fit ML models generated for all participants, 100% of participants completed intervention phase 1 (digital monitoring) and 80% of participants completed intervention phase 2 (coach guided iMAPs). Regarding non-completers, main reasons for discontinuation were their busy personal schedules that limited their participation. The 40 PerMA trial completers showed significant alleviation of depressive symptoms (PHQ9, primary outcome) at post-intervention (effect size d = −0.89, CI [−1.25, −0.53]) sustained at 6 weeks (d = −1.06, CI [−1.51, −0.6]) and 12 weeks (d = −0.52, CI [−0.95, −0.09]) follow-ups. Secondary outcomes also showed significant improvement in co-morbid anxiety (GAD7), clinician-rated depression (HDRS), quality of life (MCS12) and mindful attention awareness (MAAS). Additionally, objective cognitive outcomes significantly improved at post-intervention. Here, we discuss several nuances of these results.

The primary PHQ9 outcome and most secondary outcomes showed robust medium-to-large effect sizes for post vs. pre-intervention change. While a control group was absent in this first implementation of the PerMA approach, the effect size comparing post vs. pre-intervention results for phase 1 + 2 completers (n = 40) vs. phase 2 non-completers (n = 10) was also large (d = −0.84, CI [−1.54, −0.13]). Notably, post-hoc testing showed that PHQ9 scores significantly improved even by end of phase 1, i.e., at end of digital monitoring corresponding to the first guided session (GS1) when the coach revealed the iMAP to each participant, yet at end of phase 2, i.e., after iMAPs were successfully implemented, results were significantly better than at end of phase 1. The domain of iMAP intervention targeted to either sleep, exercise, diet or social connection did not influence outcomes suggesting that all lifestyle intervention domains were similarly effective when personalized, with the caveat that we have limited statistical power to conclude this. It was also interesting to note that social connection was the most implemented iMAP (17 of 40 participants) followed by exercise (13 of 40), which may be because these domains had a larger set of actionable variables to model than sleep (5 of 40) and diet (5 of 40) iMAPs (see Supplementary Methods section S.5 for actionable variables in each domain). Further, we did not find an influence of demographics of age, gender and ethnicity on trial outcomes. Notably, we found that improvements in quality of life (QOL), i.e., how participants felt about their psychosocial functioning and ability to derive pleasure from their life activities [80], was significantly related to improvement in depression at post-intervention as well as at follow-ups. This is aligned with evidence showing that improvements in all lifestyle domains that we targeted is associated with improvements in the QOL mental component summary measure [5, 6, 70, 81, 82]. Further, QOL improvement has been evidenced to predict lower odds of depression relapse and recurrence and thus, is a very important outcome for interventions [83].

Notably, while depression can be associated with cognitive deficits [31–37], there has been a lack of intervention studies in depression showing robust cognitive benefits. In a meta-analysis of randomized controlled trials, cognitive behavioral therapy (CBT) for depression was confirmed to benefit attention and verbal learning but no other domains of cognition [84]. There is much evidence that lifestyle intervention and adoption of healthy lifestyles promotes cognitive health and staves off cognitive decline [85–92], but again effects of personalized lifestyle optimization for cognition in depression have not been studied. Here, we show that personalized lifestyle intervention significantly improves selective attention, interference processing as well as working memory in individuals with depressive symptoms – cognitive domains that are all impacted in depression [93–96]. Moreover, these objective cognitive outcomes complement the subjective behavioral outcomes and confirm broad benefits of personalized lifestyle optimization.

We also wanted to investigate whether any behavioral/cognitive factors measured at baseline predict depression remission to healthy status. Of all measures, we found that individuals with greater mindfulness and lower anxiety scores at baseline were more likely to remit post-intervention, and these associations were significant even when controlling for baseline depression scores. Both of these factors have been linked to self-efficacy [97, 98], i.e., belief in one’s ability to make change. Mindfulness has also been shown to facilitate behavior change through greater self-awareness and control over decision-making [51, 52], which is why brief mindful attention to breathing was part of the digital schedule of the study [65]. Overall, this result suggests the importance of greater self-efficacy, via greater mindfulness and lower anxiety to effect depression remission.

Here, we also confirmed that significant change in the primary targeted lifestyle domain for each individual in intervention phase 2 relative to phase 1, measured using EMA, was significantly linked to improvement in depressed mood. This finding aligns with the strong foundation in the literature that improvements in sleep, diet and exercise are linked to improvements in depression symptoms [82, 99, 100]. This relationship emerged as early as completion of first 30% of phase 2 EMAs, signaling rapid change in personalized lifestyle behaviors associates with improved mood. Changes in off-target lifestyle domains was not significant in phase 2 and not related to improvement in depressed mood emphasizing the specificity of outcomes, i.e., the results were not driven by general lifestyle change across all domains. These results relay the importance of selective personalization of intervention to the lifestyle domain factors that best predict depressed mood in each individual.

Finally, in order to further streamline and automate the iMAP assignment, we generated a decision algorithm (DA) as well as an LLM prompt that took into account all factors of the personalized ML results, which were considered by the coaches to recommend specific iMAPs. The DA approach is transparent in that it is a simple structured equation that outputs a domain rank list for the preferred iMAP for each individual. LLM has the advantage of providing a logical explanation that may assist the coach in relaying personalized insights to the participant, yet the underlying model is a blackbox. Hence, the two approaches are complementary. Both the naïve DA and the LLM were designed a priori, i.e., with no iterations with regard to the actual iMAP assignments in the study to avoid overfitting to the dataset. These approaches achieved 87.5% (DA) and 92.5% (LLM) match with the coach’s decision, demonstrating that in a majority of cases iMAP assignment can be a straightforward process. With further empirical fine-tuning of the DA based on the actual coach iMAP assignments, the algorithmic match was improved to 95%. Yet the empirically-determined fine-tuned DA weights are overfit to our sample data and suitable only as a proof of concept. The 5% error rate of the fine-tuned DA may also be an underestimate given our modest sample size, hence, full automation without any human coach review is not yet recommended. Nevertheless, the complementary DA + LLM approaches may be useful assists for a human coach in future work.

The primary limitation of this study is the lack of a randomized control group. Lifestyle targeted behavioral interventions in depression have typically shown small-to-medium effect sizes of ~0.3–0.5 [4–16], while the placebo effect of behavioral interventions are even smaller ~0.2 in RCTs [101, 102]. The preliminary effect size of this personalized ML guided trial of 0.8 is similar to the effect size observed for CBT [103]; yet, an RCT is required for effect replication and further interpretation of the advantages of machine learning-driven personalization over non-personalization. Without an RCT, we cannot explicitly establish an effect of intervention. However, the primary purpose of this pilot study is a proof of concept to operationalize a personalized intervention pipeline. In this context, meta-analytic evidence suggests that psychotherapy personalization is an effective strategy to improve therapeutic outcomes and even small effect size advantages of personalization may have important impacts at a clinical population level [25]. In addition to a control group, a future study may leverage DA/LLM based iMAP assignment to remove potential variability introduced by human coach-based intervention assignment.

An additional limitation in this pilot study is a modest sample size of 40 completers. Although purely digital health studies might have larger sample sizes such as a similar designed behavioral health intervention for hypertension [104], our study combines aspects of digital health therapies and a one-to-one patient-coach/clinician component. When looking at hybrid human delivered pilot studies, a sample size of 40 is on par with comparable research [105, 106]. Additionally, the high within-subject data density afforded by our N-of-1 strategy serves as a compensatory strength, offering a robust level of individual insight that traditional cross-sectional studies often lack.

Despite its limitations, this trial demonstrates the promise of data-driven personalized lifestyle optimization for improving depressive symptoms, quality of life and cognition. Given the digital health implementation approach, the cost and logistics burden for participants is minimal, and such a lifestyle intervention is further devoid of side effects of pharmacotherapy [107]. An RCT as the next step will solidify the evidence-base and utility of this data-driven personalized intervention for application at scale.

## Supplementary information

Supplemental Figure

## Data Availability

De-identified and processed feature set data will be available upon request.

## References

[1] CDC. Symptoms of depression among adults: United States. 2020. *CDC Data Briefs - Number 379*. https://www.cdc.gov/nchs/products/databriefs/db379.htm 33054920

[2] Friedrich MJ. Depression is the leading cause of disability around the world. JAMA. 2017;317:1517. 28418490 10.1001/jama.2017.3826

[3] Greenberg P, Chitnis A, Louie D, Suthoff E, Chen SY, Maitland J, et al. The economic burden of adults with major depressive disorder in the United States (2019). Adv. Ther. 2023;40:4460–79. 37518849 10.1007/s12325-023-02622-x PMC10499687

[4] Sarris J, O’Neil A, Coulson CE, Schweitzer I, Berk M. Lifestyle medicine for depression. BMC Psychiatry. 2014;14:107. 10.1186/1471-244X-14-107 24721040 10.1186/1471-244X-14-107 PMC3998225

[5] Opie RS, O’Neil A, Itsiopoulos C, Jacka FN. The impact of whole-of-diet interventions on depression and anxiety: a systematic review of randomised controlled trials. Public Health Nutr. 2015;18:2074–93. 25465596 10.1017/S1368980014002614 PMC10271872

[6] Opie RS, O’Neil A, Jacka FN, Pizzinga J, Itsiopoulos C. A modified mediterranean dietary intervention for adults with major depression: dietary protocol and feasibility data from the SMILES trial. Nutr. Neurosci. 2018;21:487–501. 28424045 10.1080/1028415X.2017.1312841

[7] Hearing CM, Chang WC, Szuhany KL, Deckersbach T, Nierenberg AA, Sylvia LG. Physical exercise for treatment of mood disorders: a critical review. Curr. Behav. Neurosci. Rep. 2016;3:350–9. 28503402 10.1007/s40473-016-0089-y PMC5423723

[8] Taylor CT, Stein MB, Simmons AN, He F, Oveis C, Shakya HB, et al. Amplification of positivity treatment for anxiety and depression: a randomized experimental therapeutics trial targeting social reward sensitivity to enhance social connectedness. Biol. Psychiatry. 2024;95:434–43. 37607657 10.1016/j.biopsych.2023.07.024 PMC12063735

[9] Farah WH, Alsawas M, Mainou M, Alahdab F, Farah MH, Ahmed AT, et al. Non-pharmacological treatment of depression: a systematic review and evidence map. Evidence Based Medicine. 2016;21:214–21. 27836921 10.1136/ebmed-2016-110522

[10] Hofmann SG, Gómez AF. Mindfulness-based interventions for anxiety and depression. Psychiatric Clinics of North America. 2017;40:739–49. 29080597 10.1016/j.psc.2017.08.008 PMC5679245

[11] Noetel M, Sanders T, Gallardo-Gómez D, Taylor P, Del Pozo Cruz B, van den Hoek D, et al. Effect of exercise for depression: systematic review and network meta-analysis of randomised controlled trials. BMJ. 2024;384:e075847. 10.1136/bmj-2023-075847 38355154 10.1136/bmj-2023-075847 PMC10870815

[12] Schuch F, Vancampfort D, Firth J, Rosenbaum S, Ward P, Reichert T, et al. Physical activity and sedentary behavior in people with major depressive disorder: a systematic review and meta-analysis. J. Affect. Disord. 2017;210:139–50. 28033521 10.1016/j.jad.2016.10.050

[13] Parletta N, Zarnowiecki D, Cho J, Wilson A, Bogomolova S, Villani A, et al. A Mediterranean-style dietary intervention supplemented with fish oil improves diet quality and mental health in people with depression: a randomized controlled trial (HELFIMED). Nutr. Neurosci. 2019;22:474–87. 29215971 10.1080/1028415X.2017.1411320

[14] Francis HM, Stevenson RJ, Chambers JR, Gupta D, Newey B, Lim CK. A brief diet intervention can reduce symptoms of depression in young adults – a randomised controlled trial. PLoS One. 2019;14:e0222768. 31596866 10.1371/journal.pone.0222768 PMC6784975

[15] Carney CE, Edinger JD, Kuchibhatla M, Lachowski AM, Bogouslavsky O, Krystal AD, et al. Cognitive behavioral insomnia therapy for those with insomnia and depression: a randomized controlled clinical trial. Sleep. 2017;40:zsx019. 28199710 10.1093/sleep/zsx019 PMC5806549

[16] Hertenstein E, Trinca E, Wunderlin M, Schneider CL, Züst MA, Fehér KD, et al. Cognitive behavioral therapy for insomnia in patients with mental disorders and comorbid insomnia: a systematic review and meta-analysis. Sleep Med. Rev. 2022;62:101597. 35240417 10.1016/j.smrv.2022.101597

[17] Shah RV, Grennan G, Zafar-Khan M, Alim F, Dey S, Ramanathan D, et al. Personalized machine learning of depressed mood using wearables. Transl. Psychiatry. 2021;11:338. 34103481 10.1038/s41398-021-01445-0 PMC8187630

[18] Nan J, Herbert MS, Purpura S, Henneken AN, Ramanathan D, Mishra J. Personalized machine learning-based prediction of wellbeing and empathy in healthcare professionals. Sensors. 2024;24:2640. 38676258 10.3390/s24082640 PMC11053570

[19] Wittenborn AK, Hosseinichimeh N. Exploring personalized psychotherapy for depression: a system dynamics approach. PLoS One. 2022;17:e0276441. 36301962 10.1371/journal.pone.0276441 PMC9612473

[20] Xiao S, Gaier ED, Mazow ML, Stout AU, Travers DA, Angjeli E, et al. Improved adherence and treatment outcomes with an engaging, personalized digital therapeutic in amblyopia. Sci. Rep. 2020;10:8328. 32433490 10.1038/s41598-020-65234-3 PMC7239850

[21] Kulzer B, Daenschel W, Daenschel I, Schramm W, Messinger D, Weissmann J, et al. Integrated personalized diabetes management improves glycemic control in patients with insulin-treated type 2 diabetes: results of the PDM-ProValue study program. Diabetes Res. Clin. Pract. 2018;144:200–12. 30205184 10.1016/j.diabres.2018.09.002

[22] Linn AJ, van Dijk L, van Weert J, Gebeyehu BG, van Bodegraven AA, Smit EG. Creating a synergy effect: a cluster randomized controlled trial testing the effect of a tailored multimedia intervention on patient outcomes. Patient Educ. Couns. 2018;101:1419–26. 29609899 10.1016/j.pec.2018.03.017

[23] Chiang P-H, Wong M, Dey S. Using wearables and machine learning to enable personalized lifestyle recommendations to improve blood pressure. IEEE J. Transl. Eng. Health Med. 2021;9:1–13. 10.1109/JTEHM.2021.3098173 PMC8577573 34765324

[24] van Bronswijk SC, DeRubeis RJ, Lemmens L, Peeters F, Keefe JR, Cohen ZD, et al. Precision medicine for long-term depression outcomes using the Personalized Advantage Index approach: cognitive therapy or interpersonal psychotherapy? Psychol. Med. 2021;51:279–89. 31753043 10.1017/S0033291719003192 PMC7893512

[25] Nye A, Delgadillo J, Barkham M. Efficacy of personalized psychological interventions: a systematic review and meta-analysis. J. Consult. Clin. Psychol. 2023;91:389–97. 37166831 10.1037/ccp0000820

[26] Chekroud AM, Hawrilenko M, Loho H, Bondar J, Gueorguieva R, Hasan A, et al. Illusory generalizability of clinical prediction models. Science (1979). 2024;383:164–7. 10.1126/science.adg8538 38207039

[27] Chatterjee S, Mishra J, Sundram F, Roop P. Towards personalised mood prediction and explanation for depression from biophysical data. Sensors. 2023;24:164. 38203024 10.3390/s24010164 PMC10781272

[28] Thornicroft G, Ahuja S, Barber S, Chisholm D, Collins PY, Docrat S, et al. Integrated care for people with long-term mental and physical health conditions in low-income and middle-income countries. Lancet Psychiatry. 2019;6:174–86. 30449711 10.1016/S2215-0366(18)30298-0

[29] Hodgkinson S, Godoy L, Beers LS, Lewin A. Improving mental health access for low-income children and families in the primary care setting. Pediatrics. 2017;139:e20151175. 27965378 10.1542/peds.2015-1175 PMC5192088

[30] Rathod S, Pinninti N, Irfan M, Gorczynski P, Rathod P, Gega L, et al. Mental health service provision in low- and middle-income countries. Health Serv. Insights. 2017;10:1178632917694350. 28469456 10.1177/1178632917694350 PMC5398308

[31] Gotlib IH, Joormann J. Cognition and depression: current status and future directions. Annu. Rev. Clin. Psychol. 2010;6:285–312. 20192795 10.1146/annurev.clinpsy.121208.131305 PMC2845726

[32] Atique-Ur-Rehman H, Neill JC. Cognitive dysfunction in major depression: From assessment to novel therapies. Pharmacol. Ther. 2019;202:53–71. 31173838 10.1016/j.pharmthera.2019.05.013

[33] Chakrabarty T, Hadjipavlou G, Lam RW. Cognitive dysfunction in major depressive disorder: assessment, impact, and management. Focus (Am. Psychiatr. Publ). 2016;14:194–206. 31975803 10.1176/appi.focus.20150043 PMC6519654

[34] Remes O, Mendes JF, Templeton P. Biological, psychological, and social determinants of depression: a review of recent literature. Brain Sci. 2021;11:1633. 34942936 10.3390/brainsci11121633 PMC8699555

[35] Perini G, Cotta Ramusino M, Sinforiani E, Bernini S, Petrachi R, Costa A. Cognitive impairment in depression: recent advances and novel treatments. Neuropsychiatr. Dis. Treat. 2019;15:1249–58. 31190831 10.2147/NDT.S199746 PMC6520478

[36] Sekhon, S & Marwaha, R. *Depressive cognitive disorders*. 2025. 32644682

[37] Culpepper L, Lam RW, McIntyre RS. Cognitive impairment in patients with depression: awareness, assessment, and management. J. Clin. Psychiatry. 2017;78:1383–94. 29345866 10.4088/JCP.tk16043ah5c

[38] Kroenke K, Spitzer RL, Williams JB. The PHQ-9: validity of a brief depression severity measure. J. Gen. Intern. Med. 2001;16:606–13. 11556941 10.1046/j.1525-1497.2001.016009606.x PMC1495268

[39] First, M, Williams, JB, Spitzer, R & Gibbon, M SCID - structured clinical interview for DSM-IV axis I disorders. 2002. https://eprovide.mapi-trust.org/instruments/structured-clinical-interview-for-dsm-iv-axis-i-disorders 10.1001/archpsyc.1992.01820080032005 1637252

[40] Nasreddine ZS, Phillips NA, Bédirian V, Charbonneau S, Whitehead V, Collin I, et al. The montreal cognitive assessment, MoCA: a brief screening tool for mild cognitive impairment. J. Am. Geriatr. Soc. 2005;53:695–9. 15817019 10.1111/j.1532-5415.2005.53221.x

[41] Faul F, Erdfelder E, Buchner A, Lang A-G. Statistical power analyses using G*Power 3.1: tests for correlation and regression analyses. Behav. Res. Methods. 2009;41:1149–60. 19897823 10.3758/BRM.41.4.1149

[42] ClinicalTrials.gov NCT05662254. UCSD Depression Trial → PERsonalized Mood Augmentation Trial for Depressed Mood. https://clinicaltrials.ucsd.edu/trial/NCT05662254.

[43] Misra A, Ojeda A, Mishra J. BrainE: a digital platform for evaluating, engaging and enhancing brain function. 2018. *Regents of the University of California Copyright SD2018-816*. https://play.google.com/store/apps/details?id=com.neatlabs.braine&hl=en_US.

[44] Merikangas KR, Swendsen J, Hickie IB, Cui L, Shou H, Merikangas AK, et al. Real-time mobile monitoring of the dynamic associations among motor activity, energy, mood, and sleep in adults with bipolar disorder. JAMA Psychiatry. 2019;76:190–8. 30540352 10.1001/jamapsychiatry.2018.3546 PMC6439734

[45] Spitzer RL, Kroenke K, Williams JBW, Löwe B. A brief measure for assessing generalized anxiety disorder: the GAD-7. Arch. Intern. Med. 2006;166:1092–7. 16717171 10.1001/archinte.166.10.1092

[46] Kessler RC, Sampson NA, Berglund P, Gruber MJ, Al-Hamzawi A, Andrade L, et al. Anxious and non-anxious major depressive disorder in the World Health Organization World Mental Health Surveys. Epidemiol. Psychiatr. Sci. 2015;24:210–26. 25720357 10.1017/S2045796015000189 PMC5129607

[47] Kalin NH. The critical relationship between anxiety and depression. American Journal of Psychiatry. 2020;177:365–7. 32354270 10.1176/appi.ajp.2020.20030305

[48] Hamilton M. A rating scale for depression. J. Neurol. Neurosurg. Psychiatry. 1960;23:56–62. 14399272 10.1136/jnnp.23.1.56 PMC495331

[49] Turner-Bowker D, Hogue SJ. Short form 12 health survey (SF-12). in *Encyclopedia of quality of life and well-being research*. Springer Netherlands, Dordrecht, 2014. pp. 5954-7. 10.1007/978-94-007-0753-5_2698

[50] Brown KW, Ryan RM. The benefits of being present: mindfulness and its role in psychological well-being. J. Pers. Soc. Psychol. 2003;84:822–48. 12703651 10.1037/0022-3514.84.4.822

[51] Gawande R, To MN, Pine E, Griswold T, Creedon TB, Brunel A, et al. Mindfulness training enhances self-regulation and facilitates health behavior change for primary care patients: a randomized controlled trial. J. Gen. Intern. Med. 2019;34:293–302. 30511291 10.1007/s11606-018-4739-5 PMC6374253

[52] Schuman-Olivier Z, Trombka M, Lovas DA, Brewer JA, Vago DR, Gawande R, et al. Mindfulness and behavior change. Harv. Rev. Psychiatry. 2020;28:371–94. 33156156 10.1097/HRP.0000000000000277 PMC7647439

[53] Oquendo MA, Halberstam B, Mann J. Risk factors for suicidal behavior. The utility and limitations of research instruments. *Standardized Evaluation in Clinical Practice*. Review of Psychiatry. 2003;8:103–30.

[54] Balasubramani PP, Ojeda A, Grennan G, Maric V, Le H, Alim F, et al. Mapping cognitive brain functions at scale. Neuroimage. 2021;231:117641. 33338609 10.1016/j.neuroimage.2020.117641 PMC8221518

[55] Nan J, Grennan G, Ravichandran S, Ramanathan D, Mishra J. Neural activity during inhibitory control predicts suicidal ideation with machine learning. NPP—Digital Psychiatry and Neuroscience. 2024;2:10. 38988507 10.1038/s44277-024-00012-x PMC11230903

[56] Grennan GK, Ramanathan DS, Mishra J, Withers MC. Differences in interference processing and frontal brain function with climate trauma from California’s deadliest wildfire. PLOS Climate. 2023;2:e0000125.

[57] Mo Z, Grennan G, Kulkarni A, Ramanathan D, Balasubramani PP, Mishra J. Parietal alpha underlies slower cognitive responses during interference processing in adolescents. Behav Brain Res. 2023;443:114356. 36801472 10.1016/j.bbr.2023.114356

[58] Ramanathan D, Nan J, Grennan G, Jaiswal S, Purpura S, Manchanda J, et al. Modulation of posterior default mode network activity during interoceptive attention and relation to mindfulness. Biological Psychiatry Global Open Science. 2024;4:100384. 39416659 10.1016/j.bpsgos.2024.100384 PMC11480231

[59] Grennan G, Balasubramani PP, Alim F, Zafar-Khan M, Lee EE, Jeste DV, et al. Cognitive and neural correlates of loneliness and wisdom during emotional bias. Cerebral Cortex. 2021;31:3311–22. 33687437 10.1093/cercor/bhab012 PMC8196261

[60] Kato R, Balasubramani PP, Ramanathan D, Mishra J. Utility of cognitive neural features for predicting mental health behaviors. Sensors. 2022;22:3116. 35590804 10.3390/s22093116 PMC9100783

[61] Grennan G, Balasubramani PP, Vahidi N, Ramanathan D, Jeste DV, Mishra J. Dissociable neural mechanisms of cognition and well-being in youth versus healthy aging. Psychol. Aging. 2022;37:827–42. 36107693 10.1037/pag0000710 PMC9669243

[62] Balasubramani PP, Walke A, Grennan G, Perley A, Purpura S, Ramanathan D, et al. Simultaneous gut-brain electrophysiology shows cognition and satiety specific coupling. Sensors. 2022;22:9242. 36501942 10.3390/s22239242 PMC9737783

[63] Fakhraei L. Mapping large-scale networks associated with action, behavioral inhibition and impulsivity. eNeuro. 2021;8:ENEURO.0406-20.2021. 33509949 10.1523/ENEURO.0406-20.2021 PMC7920541

[64] Fakhraei L, Francoeur M, Balasubramani PP, Tang T, Hulyalkar S, Buscher N, et al. Electrophysiological correlates of rodent default-mode network suppression revealed by large-scale local field potential recordings. Cereb. Cortex Commun. 2021;2:034. 10.1093/texcom/tgab034 PMC8166125 34296178

[65] Jaiswal S, Nan J, Dizon S, Young JO, Purpura SR, Manchanda JK, et al. Breath-focused mindfulness and compassion training in parent-child dyads: Pilot Intervention Study. JMIR Form. Res. 2025;9:e69607. 40674736 10.2196/69607 PMC12289298

[66] Jaiswal S, Purpura SR, Manchanda JK, Nan J, Azeez N, Ramanathan D, et al. Design and Implementation of a brief digital mindfulness and compassion training app for health care professionals: cluster randomized controlled trial. JMIR Ment. Health. 2024;11:e49467. 38252479 10.2196/49467 PMC10845023

[67] Molnar C. 5.9 shapley values | interpretable machine learning. in *Interpretable Machine Learning*. 2020.

[68] Asarnow LD, Manber R. Cognitive behavioral therapy for insomnia in depression. Sleep Med. Clin. 2019;14:177–84. 31029185 10.1016/j.jsmc.2019.01.009 PMC6487874

[69] Sallis R. Exercise is medicine: a call to action for physicians to assess and prescribe exercise. Phys. Sportsmed. 2015;43:22–6. 25684558 10.1080/00913847.2015.1001938

[70] Taylor CT, Lyubomirsky S, Stein MB. Upregulating the positive affect system in anxiety and depression: outcomes of a positive activity intervention. Depress. Anxiety. 2017;34:267–80. 28060463 10.1002/da.22593 PMC7266488

[71] Cohen J. *Statistical power analysis for the behavioral sciences*. Routledge; 2013. 10.4324/9780203771587.

[72] Lane K. *What Is Robust Regression and How Do You Do It?* (2002).

[73] Google. Gemini developer API. 2024. https://ai.google.dev/gemini-api/docs

[74] Heinz MV, Mackin DM, Trudeau BM, Bhattacharya S, Wang Y, Banta HA, et al. Randomized trial of a generative AI chatbot for mental health treatment. *NEJM AI* 2025;2. 10.1056/AIoa2400802

[75] Siddals S, Torous J, Coxon A. ‘It happened to be the perfect thing’: experiences of generative AI chatbots for mental health. Npj mental health research. 2024;3:48. 39465310 10.1038/s44184-024-00097-4 PMC11514308

[76] Farzan M, Ebrahimi H, Pourali M, Sabeti F. Artificial intelligence-powered cognitive behavioral therapy chatbots, a systematic review. Iran. J. Psychiatry. 2025;20:102–10. 40093525 10.18502/ijps.v20i1.17395 PMC11904749

[77] Yonatan‐Leus R, Brukner H. Comparing perceived empathy and intervention strategies of an AI chatbot and human psychotherapists in online mental health support. *Couns. Psychother. Res*. 2025;25. 10.1002/capr.12832

[78] Spytska L. The use of artificial intelligence in psychotherapy: development of intelligent therapeutic systems. BMC Psychol. 2025;13:175. 40022267 10.1186/s40359-025-02491-9 PMC11871827

[79] Khawaja Z, Bélisle-Pipon J-C. Your robot therapist is not your therapist: understanding the role of AI-powered mental health chatbots. Front. Digit. Health. 2023;5:1278186. 38026836 10.3389/fdgth.2023.1278186 PMC10663264

[80] Papakostas GI, Petersen T, Mahal Y, Mischoulon D, Nierenberg AA, Fava M. Quality of life assessments in major depressive disorder: a review of the literature. Gen. Hosp. Psychiatry. 2004;26:13–17. 14757297 10.1016/j.genhosppsych.2003.07.004

[81] Nicolucci A, Haxhi J, D'Errico V, Sacchetti M, Orlando G, Cardelli P, et al. Effect of a behavioural intervention for adoption and maintenance of a physically active lifestyle on psychological well-being and quality of life in patients with type 2 diabetes: the IDES_2 randomized clinical trial. Sports Medicine. 2022;52:643–54. 34599476 10.1007/s40279-021-01556-0 PMC8891112

[82] Scott AJ, Webb TL, Martyn-St James M, Rowse G, Weich S. Improving sleep quality leads to better mental health: a meta-analysis of randomised controlled trials. Sleep Med. Rev. 2021;60:101556. 34607184 10.1016/j.smrv.2021.101556 PMC8651630

[83] Vittengl JR, Jha MK, Minhajuddin A, Thase ME, Jarrett RB. Quality of life after response to acute-phase cognitive therapy for recurrent depression. J. Affect. Disord. 2021;278:218–25. 32971314 10.1016/j.jad.2020.09.059 PMC7704560

[84] Liu L, Wang K, Xu D, Wang Y, Xu X, Wang Q, et al. Effectiveness of cognitive rehabilitation in improving symptoms and restoring cognitive functions in patients with depression: an updated meta-analysis of randomized controlled trials. Alpha psychiatry. 2024;25:727–36. 39830048 10.5152/alphapsychiatry.2024.241731 PMC11736863

[85] Ngandu T, Lehtisalo J, Korkki S, Solomon A, Coley N, Antikainen R, et al. The effect of adherence on cognition in a multidomain lifestyle intervention (FINGER). Alzheimer’s & Dementia. 2022;18:1325–34. 10.1002/alz.12492 34668644

[86] Balsamo F, Berretta E, Meneo D, Baglioni C, Gelfo F. The complex relationship between sleep and cognitive reserve: a narrative review based on human studies. Brain Sci. 2024;14:654. 39061395 10.3390/brainsci14070654 PMC11274941

[87] Livingston G, Huntley J, Sommerlad A, Ames D, Ballard C, Banerjee S, et al. Dementia prevention, intervention, and care: 2020 report of the lancet commission. Lancet. 2020;396:413–46. 32738937 10.1016/S0140-6736(20)30367-6 PMC7392084

[88] Zou C, Amos-Richards D, Jagannathan R, Kulshreshtha A. Effect of home-based lifestyle interventions on cognition in older adults with mild cognitive impairment: a systematic review. BMC Geriatr. 2024;24:200. 38413870 10.1186/s12877-024-04798-5 PMC10900825

[89] Naaman RK, Alashmali S, Bakhsh MA, Alneami SA, Algamdi ES, Al-Ghamdi GA, et al. Association between healthy lifestyle and cognitive function in middle-aged and older adults. Healthcare. 2025;13:1140. 40427976 10.3390/healthcare13101140 PMC12110782

[90] Vidyanti AN, Rahmawati F, Rahman RH, Prodjohardjono A, Gofir A. Lifestyle interventions for dementia risk reduction: A review on the role of physical activity and diet in Western and Asian Countries. J. Prev. Alzheimers Dis. 2025;12:100028. 39863321 10.1016/j.tjpad.2024.100028 PMC12184051

[91] Ding Z, Leung P-Y, Lee T, Chan AS. Effectiveness of lifestyle medicine on cognitive functions in mild cognitive impairments and dementia: a systematic review on randomized controlled trials. Ageing Res. Rev. 2023;86:101886. 36806378 10.1016/j.arr.2023.101886

[92] Baker LD, Espeland MA, Whitmer RA, Snyder HM, Leng X, Lovato L, et al. Structured vs self-guided multidomain lifestyle interventions for global cognitive function. JAMA. 2025;334:681–91. 40720610 10.1001/jama.2025.12923 PMC12305445

[93] Keller AS, Leikauf JE, Holt-Gosselin B, Staveland BR, Williams LM. Paying attention to attention in depression. Transl. Psychiatry. 2019;9:279. 31699968 10.1038/s41398-019-0616-1 PMC6838308

[94] Nikolin S, Tan YY, Schwaab A, Moffa A, Loo CK, Martin D. An investigation of working memory deficits in depression using the n-back task: a systematic review and meta-analysis. J. Affect. Disord. 2021;284:1–8. 33581489 10.1016/j.jad.2021.01.084

[95] Fales CL, Barch DM, Rundle MM, Mintun MA, Snyder AZ, Cohen JD, et al. Altered emotional interference processing in affective and cognitive-control brain circuitry in major depression. Biol. Psychiatry. 2008;63:377–84. 17719567 10.1016/j.biopsych.2007.06.012 PMC2268639

[96] Kaiser RH, Andrews-Hanna JR, Spielberg JM, Warren SL, Sutton BP, Miller GA, et al. Distracted and down: neural mechanisms of affective interference in subclinical depression. Soc. Cogn. Affect. Neurosci. 2015;10:654–63. 25062838 10.1093/scan/nsu100 PMC4420741

[97] Sharma PK, Kumra R. Relationship between mindfulness, depression, anxiety and stress: mediating role of self-efficacy. Pers. Individ. Dif. 2022;186:111363.

[98] Martínez-Pérez I, García-Rodríguez A, Morales-Rodríguez FM, Pérez-Mármol JM. Mindfulness abilities are associated with anxiety levels, emotional intelligence, and perceived self-efficacy. Sustainability. 2023;15:4729.

[99] Moradi F, Heshmati J, Daneshzad E, Ahmadi A, Jafari T, Persad E, et al. Association between dietary satisfaction and depression, anxiety and stress in obese and overweight patients during the coronavirus pandemic. Clin. Nutr. ESPEN. 2021;45:399–403. 34620346 10.1016/j.clnesp.2021.07.013 PMC8299144

[100] Craft LL, Perna FM. The benefits of exercise for the clinically depressed. Prim. Care Companion CNS Disord. 2004;6:104–11. 10.4088/pcc.v06n0301 PMC474733 15361924

[101] Fernández‐López R, Riquelme‐Gallego B, Bueno‐Cavanillas A, Khan KS. Influence of placebo effect in mental disorders research: a systematic review and meta‐analysis. Eur. J. Clin. Invest. 2022;52:13762. 10.1111/eci.13762 PMC9286474 35224726

[102] Lindheimer JB, O’Connor PJ, Dishman RK. Quantifying the placebo effect in psychological outcomes of exercise training: a meta-analysis of randomized trials. Sports Medicine. 2015;45:693–711. 25762083 10.1007/s40279-015-0303-1

[103] Cuijpers P, Miguel C, Harrer M, Plessen CY, Ciharova M, Ebert D, et al. Cognitive behavior therapy vs. control conditions, other psychotherapies, pharmacotherapies and combined treatment for depression: a comprehensive meta‐analysis including 409 trials with 52,702 patients. World Psychiatry. 2023;22:105–15. 36640411 10.1002/wps.21069 PMC9840507

[104] Leitner J, Chiang P-H, Agnihotri P, Dey S. The effect of an AI-based, autonomous, digital health intervention using precise lifestyle guidance on blood pressure in adults with hypertension: single-arm nonrandomized trial. JMIR Cardio. 2024;8:e51916. 38805253 10.2196/51916 PMC11167324

[105] Tang Y, Gierc M, La H, Liu S, Lam RW, Puterman E, et al. Feasibility and preliminary effects of an app-based physical activity intervention for individuals with depression (MoodMover): a protocol for a single-arm, pre-post intervention study. PLoS One. 2025;20:e0321958. 40261856 10.1371/journal.pone.0321958 PMC12013873

[106] Billingham SAM, Whitehead AL, Julious SA. An audit of sample sizes for pilot and feasibility trials being undertaken in the United Kingdom registered in the United Kingdom Clinical Research Network database. BMC Med. Res. Methodol. 2013;13:104. 23961782 10.1186/1471-2288-13-104 PMC3765378

[107] Braund TA, Tillman G, Palmer DM, Gordon E, Rush AJ, Harris A. Antidepressant side effects and their impact on treatment outcome in people with major depressive disorder: an iSPOT-D report. Transl. Psychiatry. 2021;11:417. 34349116 10.1038/s41398-021-01533-1 PMC8338944

