# Supplementary material for: Personalized machine learning guided intervention for optimizing lifestyle behaviors in depression: a pilot study
Source: NPP Digit Psychiatry Neurosci. 2026 May 19;4:10. doi: 10.1038/s44277-026-00062-3 (PMC13184138; doi:10.1038/s44277-026-00062-3)
Supplement: Supplementary file 1 — Supplemental Figure [file 44277_2026_62_MOESM1_ESM.docx]

**Supplementary Materials**

**Supplementary Methods**

**S.1 Cognitive Assessments.** At pre and post-intervention, participants engaged in ~30-minutes of cognitive assessments on the BrainE© platform. Participants engaged in four assessments of (1) selective attention, (2) interference processing, (3) working memory, and (4) emotion bias, and had the opportunity to take self-paced breaks between tasks to minimize fatigue. Tasks were run in the same order for all participants.

**Supplementary figure 1** shows the stimulus sequence in each task. All four cognitive tasks had a standard trial structure of 500 ms central fixation “+” cue followed by a task-specific stimulus presented for task-specific duration and with a task-specific response window. This response window in each task was adaptive with a 3up-1down staircase scheme that maintains accuracy at ~80% and engages the user by avoiding ceiling performance ^1,2^. An adaptive scheme also reduces practice effects that affect repeat assessment sessions. Further details of the adaptive scheme in each task are provided below.

Supplementary Figure 1. Cognitive assessments delivered on the BrainE platform. At top left, the BrainE assessment dashboard is shown. (A) Selective attention was measured on the ‘Go’ trials of the Go Wait task that required rapid and accurate responses to blue rocket targets. All other color rockets were non-targets on which participants waited to respond for up to 2 sec. (B) In the Flanker interference processing Middle Fish task, flanking fish may either face the same direction as the middle fish on congruent trials as shown, or the opposite direction on incongruent trials; participants were instructed to respond to the direction of the middle fish. (C) The visuo-spatial working memory task, Lost Star, was presented with perceptually thresholded stimuli and participants responded after a working memory period whether a probe star was positioned in one of the same locations as the prior test star stimuli. (D) The emotion bias task, Face Off presented neutral, happy, sad, or angry faces superimposed on an arrow, whose direction was discriminated by participants. The likeness of the person is from the Nim-Stim database^3^.

Stimuli in each cognitive task were presented in a shuffled order across trials. Response in every task trial was followed by standard response feedback for accuracy as a smiley or sad face emoticon, presented 200 ms post-response for 200 ms duration, followed by a 500 ms inter-trial interval (ITI). ITI jitter within tasks was not applied to keep the task administration rapid. At the end of each task block, participants received a percent block accuracy score with a series of happy face emoticons (up to 10) to promote engagement.

(1) Selective Attention: Participants accessed a game-like task, Go Wait modeled after the standard test of variables of attention ^4^. On each task trial, colored rockets were presented in the upper or lower central visual field. Participants were instructed to respond as rapidly as possible to blue colored rocket targets and wait to respond for 2 sec to distracting rockets of five other iso-luminant colors (shades of brown, teal, pink, purple). Iso-luminant colors were ensured using luminosity measurements in Photoshop. Luminance values for iso-luminant stimuli were 128, estimated as 0.30*R+0.59*G+0.11*B (where RGB are the Red/Green/Blue values of the chosen stimulus color). Post-fixation cue, a target/non-target stimulus appeared for 100 ms duration. For the blue rocket targets, the initial response window was set at 700 ms that adapted on each trial in a 3up-1down scheme, i.e., the response window reduced -33 ms after correct trials and increased +100 ms after incorrect trials. One happy face emoticon followed correct trials. If response was very rapid within 100-400 ms, then two happy face emoticons were presented for feedback to reinforce fast and accurate responding ^5^. For nontarget rockets, response times were not adaptive; participants waited for 2 sec at which time the fixation cue flashed briefly for 100 ms and then participants responded. Across two blocks, target and non-target trials were shuffled with 50% probability for a total of 180 trials. Response efficiency, i.e., the product of accuracy and speed was taken as the main selective attention outcome measure ^6,7^; here, accuracy was measured as the signal detection sensitivity, d’, computed as z(Hits)-z(False Alarms) ^8^ and task speed was calculated as log(1/RT), where RT is the average response time across attended trials in milliseconds.

(2) Interference Processing: Participants accessed the game-like task, Middle Fish, an adaptation of the Flanker assessment ^9–11^. Post-fixation on each trial, participants viewed an array of fish presented in the upper or lower central visual field for 100 ms. On each trial, participants had up to a 1 sec response window to detect the direction of the middle fish in the set (left or right) while ignoring the flanking distractor fish that were either congruent or incongruent to the middle fish, i.e., faced the same or opposite direction to the middle fish. Response windows were adapted on congruent trials in a 3up-1down scheme (-33 ms after correct trials and +100 ms after incorrect trials) and incongruent trial response windows matched that of the previous congruent trial. The fish flanking the middle fish interfere with the discrimination of the direction of the middle fish (left/right), hence, the task assesses interference processing. Task trials were shuffled with congruent/incongruent distractors in 1:1 ratio in 96 trials over two blocks. As in the selective attention task, response efficiency, i.e., the product of accuracy (i.e., signal detection sensitivity: d’) and speed was taken as the main outcome measure.

(3) Working Memory: Participants accessed a game-like task, Lost Star, which was based on the visuo-spatial Sternberg task ^12^. Post-fixation cue on each trial, participants viewed a spatially distributed test array of objects (i.e., a set of blue stars) for 1 sec. Participants were required to maintain the locations of these stars for a 3 sec delay period utilizing their working memory. A probe object (a single green star of 1 sec duration) was then presented in either the same spot as one of the original test stars, or in a different spot than any of the original test stars. The participant was instructed to respond whether the probe star had the same or different location as one of the test stars. 50% of task trials had the same probe star location as one of the test stars while 50% had different location, and presented in shuffled order. For each participant, we implemented this task at the threshold perceptual span, which was defined by the number of test star objects that the individual could correctly encode without any working memory delay. For this, a brief perceptual thresholding period preceded the main working memory task, allowing for equivalent perceptual load to be investigated across participants ^10^. During thresholding, the set size of test stars increased progressively from 1-8 stars based on accurate performance where 100% accuracy led to an increment in set size; <100% performance led to one 4-trial repeat of the same set size and any further inaccurate performance aborted the thresholding phase. The final set size at which 100% accuracy was obtained was designated as the individual’s perceptual threshold. Post-thresholding, the working memory task presented 48 trials over two blocks. Unlike the two previous tasks, this task was not speeded but instead adapted the working memory period in a 3up-1down scheme; if correct, the working memory period increased by +0.9 sec or if incorrect, it decreased by -0.3 sec. As in the above two tasks, response efficiency was taken as the main outcome measure but also weighted by each person’s item span, i.e., the product of item span, accuracy (i.e., signal detection sensitivity: d’) and speed was taken as the main outcome measure.

(4) Emotion Bias: Participants accessed the game-like assessment, Face Off, adapted from studies of attentional bias in emotional contexts ^13,14^ The task integrated a standardized set of culturally diverse faces from the NimStim database ^3^. We used an equivalent number of male and female faces, each face with four sets of emotions: neutral, positive (happy), negative (sad) or threatening (angry), presented on equivalent number of trials in each task block. Post-fixation cue on each trial, participants viewed an emotional face with a superimposed arrow of 300 ms duration. The arrow occurred in either the upper or lower central visual field on equal number of trials. Participants responded to the direction of the arrow (left/right) within an ensuing 1 sec response window. For neutral emotion trials, this response window adapted in a 3up-1down scheme (-33 ms after correct trials and +100 ms after incorrect trials). All other emotion trials followed the same response window as their previous neutral emotion trial. This task evaluates emotion bias (or interference) as the emotional faces interfere with the discrimination of the direction (left/right) of the arrow on which they are superimposed. Participants completed 144 trials presented over three equipartitioned blocks. Again, response efficiency, i.e., the product of overall accuracy and speed was monitored as the main outcome measure.

**S.2 Ecological Momentary Assessments.** EMAs were used to monitor mood ratings and lifestyle factors including sleep, diet, exercise, and social connection as per our prior publications ^15,16^ and as detailed below –

Mood Ratings: Participants rated depression and anxiety on 14-point Likert scales shown as green to red color gradient scales. For depression, participants responded to “How happy vs. sad/ depressed are you feeling?” with the “Happy” label anchor next to score of 1 in light green and the “Sad or Depressed” label anchor next to score of 14 in red. For anxiety, participants responded to “How relaxed vs. anxious are you feeling?” with the “Relaxed” label anchor next to score of 1 in light green and the “Anxious” label anchor next to score of 14 in red.

Sleep EMA: Only at their first EMA each day, participants reported their prior night’s sleep time, wake up time, sleep duration, percentage estimate of the time in bed spent asleep (sleep efficiency), and provided a 1–5-star rating of their sleep satisfaction. At EMAs every other time in the day, participants reported nap duration in minutes if they had napped in the past 4 hours.

Diet EMA: At each EMA, participants reported on their recent consumption of sugars, fats, and caffeine in the past 4 hours. To improve compliance, we opted for a simplified version of diet reporting instead of more objective methodologies which can be burdensome ^17,18^. Specifically, within the context of depression, excessive consumption of processed fats and sugars has been related to the severity of symptoms, and intervention to change such diet patterns has shown success ^19–22^. Hence, based on a standard assessment of dietary fats and sugars ^23^, participants responded how many portions (0-12) of each of the following items they had consumed in the past 4 hours: Red meat burger/sandwich, sausage/salami/bacon, whole egg, white bread, pizza, cheese, french fries, chips, butter popcorn, whole milk/milkshake, and fast-food take-out (fats category); cake/cookies, ice-cream, chocolate, candy, pancakes/french toast, jam/honey, soda, juice or other sweetened beverage, and cereal with added sugar (sugars category); and cups of caffeine (coffee/tea/energy drink). Participants also provided a 1-5 star rating of their diet satisfaction.

Exercise EMA: At each EMA, participants reported the amount of exercise (in hours and minutes) they did in the past 4 hours, if any, in these three categories:

1. Strenuous exercise (e.g., running, vigorous sports or bicycling)
2. Moderate exercise (e.g., fast walking, easy bicycling, swimming, dancing)
3. Mild exercise (e.g. yoga, easy walking)

Participants also provided a 1-5 star rating of their exercise satisfaction.

Social Connection EMA: At each EMA, participants reported their social connection in the past 4 hours:

1. Did you chat with family/friends? Yes/No

If yes, how many people close to you did you talk to? 1-10+

how much total time did you spend chatting (in hours and minutes)?

1. Did you attend an organized group in-person or online? (support/sports/exercise/hobby/professional group)

If yes, how long were you engaged (in hours and minutes)?

1. Did you do volunteer work for any organization in-person or online (religious, charitable, political, health-related)?

If yes, how long were you engaged (in hours and minutes)?

Participants also provided 1-5 star rating of their social connection satisfaction.

**S.3 Daily mindful awareness.** At end of every first of four EMAs during the digital monitoring period, participants completed 5 minutes of daily mindful attention to breathing ^24,25^, entered a positive self-reflection and rated their gratitude; these brief practices were employed to engage mindful awareness. In addition, after every three of four EMAs, participants completed a 30-second stress assessment with attention to breathing. These brief practices were employed to engage mindful awareness and build a foundation to support behavior change in phase 2 ^26–32^

The daily mindful attention to breathing was delivered in a closed-loop game-like format, was performance adaptive and allowed for moment-to-moment performance tracking for quantifying progress and adherence during each session ^33–35^. Specifically, individuals were requested to close their eyes, pay attention to their breathing, and tap the mobile screen after a specific number of breaths. The app monitored the consistency of tap responses. If the user was distracted based on the low consistency of breath monitoring taps, a gentle chime reminded the user to let go of the distraction and revert their attention back to mindful breathing. Initially, at level 1, participants tapped the screen after each breath. If they were able to do this consistently they graduated to tracking 2 breaths at a time, and so on until they were monitoring max 10 breaths at a time at level 10. When the practice ended and participants opened their eyes, they would see a peaceful nature scene slowly unfold as a form of training reward.

For the brief positive self-reflection, participants were prompted with a simple question based on the literature of positive psychology and gratitude ^36^. Question prompts were refreshed every few sessions and included: (1) Who or what made you smile? (2) Who or what are you thankful for? (3) Note a moment you enjoyed. (4) Note an act of kindness you did or observed. (5) Note a moment you found inspiring. (6) Who or what keeps you going? (7) Note a moment worth celebrating! (8) Everyone has personal strengths. Recognize one of yours. (9) Think of a challenge you faced, small or big, and what you learned from it. (10) Dedicate a note of appreciation to yourself or your loved one(s). Participants completed a brief text response and total time spent on the module as well as active time spent typing out a response was recorded.

Gratitude was rated on a 1-7 Likert scale as a response to the prompt, “Take a moment to indicate how grateful you are feeling.”; participants were given 7 icons graded from rainy weather to sunny weather to choose from.

For the 30-second stress assessment, participants simply tapped the mobile screen after each full breath (inhale plus exhale) ^15^. Recent research shows that consistency of tapping on this basic assay inversely relates to the internally distracted/ruminative state of the individual, which is exacerbated in depression ^37^. Mean breathing response time and consistency data were extracted on this assessment.

**S.4 Intervention Phase 1 - iMAP Modeling.** The modeling pipeline is shown in Supplementary Figure 2 below. The first step prior to ML modeling involved data ingestion, feature extraction and data preprocessing. For this the data features were extracted from each participant’s EMA, mindful awareness module and smartwatch data as below:

EMA data -

1. Time of the day when a particular dependent variable (DV, i.e., depressed mood rating) was taken: (0:00, 10:00), (10:00, 14:00), (14:00, 18:00), (18:00, 23:59)
2. Anxiety ratings completed at each time point when a DV rating was obtained.
3. Sleep time, wake up time, sleep duration, percent estimate of time in bed spent asleep, and 1–5-star rating on sleep satisfaction the previous night.
4. Exercise duration for each intensity type and total satisfaction in the 24 h period prior to each DV rating.
5. Total amount of fats, sugars, caffeine, and diet satisfaction in the last 24 h of each DV rating.
6. Number of people and total time spent chatting, total time in an organized group, total time spent volunteering, and total satisfaction in the 24 h period prior to each DV rating.

Mindful awareness module data –

1. Attention to breathing mean time and consistency obtained at each DV rating.
2. Gratitude rating completed every fourth DV rating.
3. Total and active response time in the positive self-reflection completed every fourth DV rating.

Smartwatch data –

1. Heart rate taken as the mean value within a ±30 min window around the time of each DV rating.
2. Cumulative step features taken as the mean values from the past 12 h of each DV rating for each step feature separately, i.e., count, speed, distance and calories burned.
3. Cumulative exercise features taken as the mean values from the past 12 h of each DV rating calculated for each feature separately, i.e., duration and calories burned.

Features which participants had no responses for through the entire study were considered missing and dropped for that participant. All features were calculated and stored separately for each subject for a max of 34 possible features per participant. Data were also inspected using both automated and manual approaches for unusable and missing variables, as well as variables with zero variance that were dropped for that participant. We did not implement any additional feature selection such as principal component analysis, which would dissociate variables from their physical attributes, to preserve model interpretability. Manual inspection of EMA data was also done in cases where data was not delimited properly, and to correct any data values that were saved in the wrong units (i.e., using hour fractions instead of hour and minute representations for durations). Other manual inspection of raw data was only used to verify meta data file names, variable names, and data format differences that occurred from different mobile operating systems and smartwatch versions.

Data preprocessing took participant data matrices from the prior step for purposes of imputation, standardization, and regularization. The preprocessing ensured to not alter the overall distribution of the data at the level of each participant. Iterative imputation was used for any missing data. For personalized models, removing missing data can create unaccountable bias and lead to low accuracy on test data. Moreover, filling missing values with fixed values, mean, mode, or median can also cause problems; when filled in place of missing data, these values can alter the original multivariate distribution, which may hinder the model from generalizing actual patterns in the training dataset. Thus, for missing data, we used iterative imputation, a regression-based multivariate imputation scheme ^38^. This scheme models each feature with missing values as a function of other features and uses that estimate for imputation. It does so in an iterative round-robin fashion: at each step, a feature column is designated as output Y, and the other feature columns are treated as inputs X. A regressor is fit on (X, y) for known Y. Then, the regressor is used to predict the missing values of y, executed for each feature in an iterative fashion. This iterative imputer is referred to as Multivariate Imputations via Chained Equations (MICE) ^15,16^.

To achieve preprocessing efficiency over computationally heavy ML processes, a preprocessing pipeline object was used. Using such an object has various advantages, including but not limited to encapsulating the preprocessing steps together, and avoiding leaking statistics from the test data into the trained model in cross-validation (CV) by ensuring that the same samples are used to train the transformers and predictors, and improving run time during parallel processing. For this study, the following preprocessing pipeline strategy was devised: (a) continuous and discrete variables were processed independently, (b) discrete variables were imputed using a "most frequent class imputer", which is essentially filling missing values with the class with highest frequency, (c) the continuous variables were imputed using the MICE method described above, (d) all discrete variables were regularized using an ordinal encoder, which results in a single column of integers (0 to n-categories - 1) per feature, and finally (e) all continuous variables were regularized using a maximum absolute scaler, which scales and translates each feature individually with the maximum absolute value in the training set such that it does not shift or center the data, thereby, not destroying any sparsity. The data was then ready to be deployed in the ML analysis pipeline.

ML Pipeline. A primary step to achieving robust ML models is ensuring independence between training and test, and providing transparency on the models that are evaluated. The personalized ML pipeline included hyperparameter tuning, model training, evaluation, and model selection. On the one hand, ensuring independence between data, which is used for hyperparameter tuning, training and testing makes the model less prone to overfitting, and prevents the introduction of bias into the model. However, ensuring independence between training and test datasets is a particular challenge for the N-of-1 modeling approach. A traditional k-fold cross-validation (CV) scheme cannot be used in this case as the model performance would then be highly dependent on the small number of examples set aside for testing. Thus, to tackle this technical challenge and achieving a model practically free from bias and immune to overfitting, a nested CV scheme was used ^39–43^. Specifically, we used a repeated 4-fold CV scheme with ten repeats as the inner CV strategy and a simple 4-fold CV scheme as the outer CV strategy for the overall nested CV scheme. We then modeled individual depression ratings based on the various EMA and smartwatch lifestyle predictors, employing supervised ML regression models hyperparameter tuned and trained over the nested CV scheme. The main steps of the pipeline include comparison of multiple ML strategies for each subject including Random Forest, Gradient Boost, Adaptive (Ada) Boost, Elastic Net, Support Vector, Poisson Regressor and Long Short-term Memory. The Voting Regressor employed the best model from all the other ML strategies besides LSTM. After hyperparameter tuning and training over all ML models, results were evaluated for each model, and each subject over the regression metrics of mean absolute percentage error (MAPE). We used MAPE as the performance metric to choose the best model (with lowest error) for each ML strategy. The best model for each strategy was then fed in the voting regressor. The best model from this strategy was calculated in the same manner as the other strategies. We then compared the outcome of the best performing models from each ML strategy and calculated the overall best model with the least overall MAPE. Thus, each study participant would have their own personalized model predicting their depressed mood fluctuations over time.

**Supplemental Figure 2.** Flow of the personalized Machine Learning (ML) modeling pipeline ^15,16^.

**S.5 Intervention Phase 2 – coach guided iMAPs.** Behavioral coaches reviewed the personalized ML Shapley results to determine the top-priority lifestyle intervention domain (i.e., sleep/ exercise/ diet/ social connection) for each iMAP.

Sleep iMAP. This plan was chosen if low sleep efficiency and low sleep satisfaction (actionable features in the sleep domain) were top predictors of an individual’s depressed mood. The sleep iMAP is based on cognitive behavioral therapy for insomnia (CBT-I) that has shown medium effect size improvements (d=0.5, confidence interval = 0.3-0.8) for depression treatment ^44,45^. Guided session (GS) 1 focuses on review of the personalized ML model to show top-ranking sleep predictors for depressed mood. The health coach then introduces the concepts of CBT-I including sleep hygiene and stimulus control, waking up at a regular time and avoiding/reducing naps. Participants keep daily electronic sleep logs that are then reviewed each week by the health coach. In GS2, the concept of sleep efficiency is introduced and the coach reviews whether the participant is keeping up with sleep hygiene guidelines. In GS3, daily sleep log review continues and if high sleep efficiency is achieved, then time in bed is incrementally increased. The participant also completes a SMART (Specific and small, Measurable, Action oriented, Realistic, Time stamped) goals assessment for sleep hygiene. GS4 focuses on thoughts around sleep and encourages scheduling worry time during the day (not in bed). It also introduces practicing cognitive restructuring around negative sleep thoughts (Example: “If I can’t get a good night’s sleep my day tomorrow will be shot”; Restructured: “Even if I don’t sleep well tonight, I can still get up in the morning and do things. And the more active I am tomorrow, the easier it will be to fall asleep tomorrow night.”). GS5 is dedicated to continued review of the daily sleep log, any difficulty with sleep hygiene and restructuring sleep thoughts, and titrating time in bed based on sleep efficiency over the past week. GS6 continues to review prior sleep log progress and focuses on relapse prevention by assigning an action plan to address insomnia in the future.

Exercise iMAP: This plan was chosen if exercise features were top predictors of an individual’s depressed mood. Actionable feature variables in the exercise domain included cumulative step distance, cumulative step speed, cumulative step calories, cumulative step rate, heart rate, exercise satisfaction, exercise calories, exercise duration, past day mild/moderate/strenuous exercise. Like the sleep intervention, exercise has also shown moderate effect size improvements in depression per systematic review and meta-analysis of exercise randomized control trials (RCTs) ^46^. In this iMAP, participants first review their personalized ML model for daily exercise features as top-ranking predictors of depressed mood. In GS1, the coach discusses what a consistent exercise training plan would look like for the participant. The coach uses a motivational interviewing strategy to discuss where participants are at in their change process (pre-contemplative, contemplative, ready for action) and identify specific obstacles that have prevented change in the past. As 150 minutes/week of exercise are recommended for general health ^47^, the program’s goal are to help participants to progressively build up to that level. At GS1, the participant is also requested to completed a SMART exercise solutions assignment in which the participant identifies challenges to regular exercise and feasible solutions to these. In GS2-6, the coach reviews the participant’s exercise EMA and smartwatch logs as well as SMART exercise solutions. The coach encourages and amplifies positive progress made and discusses any revisions to the SMART exercise solutions, if necessary. The participant is encouraged to increase exercise target goals by 20% each week until participant reaches about 150 mins/week of consistent activity. This activity is also objectively monitored with smartwatch data.

Diet iMAP: This plan was chosen based on diet variables, including diet satisfaction, past day fats, and past day sugars (actionable features in the diet domain) as top predictors of depressed mood. This plan is based on the modified Mediterranean diet that has shown evidence for efficacy in depression alleviation ^48,49^. In GS1, the coach reviews the participant’s personalized ML dietary predictors of depressed mood and general patterns of poor eating and diet dissatisfaction. Similar to the exercise plan, motivational interviewing is used to identify barriers to diet change, and that the goal is to re-align eating as close as possible to the target healthy mood diet. Contents of the healthy mood diet are introduced including servings of whole grains, fruits and vegetables, nuts, legumes, fish, eggs and olive oil. The diet recommends to reduce dairy, red meat and poultry servings and avoid sweets, processed cereal, chips, pastries, fried food, fatty meat, dairy, desserts, sugary drinks, condiments and alcohol. Weekly eating tips are provided along with convenient meal and snack ideas and example meal plans. A healthy mood diet grocery shopping list is also provided. Participants are encouraged to complete a healthy mood diet log that is reviewed weekly. In session 2-6, the coach reviews the participant’s healthy mood diet log and the participant completes a SMART eating solutions assignment. The coach also discusses how tangible change can be progressively achieved, for instance, focusing on changing one meal a week (i.e. breakfast/lunch/snack/dinner) instead of all meals at once. The final session focuses on maintaining the consistent progress made with the healthy mood dietary changes.

Social Connection iMAP: This plan was chosen if number of people and total time spent chatting (connect chat people and chat time features), total time in an organized group (connect group time), total time spent volunteering (connect volunteer time), connection satisfaction, gratitude and active reflection time (all actionable features in the social domain) appeared as top predictors of depressed mood. This iMAP is based on the behavioral intervention for positive amplification of mood that has shown improvements in positive affect and social connectedness ^50,51^. The intervention comprises 3 core elements: 1) increasing exposure and responsiveness to positive events; 2) practicing gratitude; and 3) engaging in kind/ generous acts toward others. In GS1, the coach reviews the participant’s personalized ML and specific social connection predictors of depressed mood, and introduces the three core elements of the positive amplification of mood plan. The participant is given a goal setting assignment to note down what’s important to them in life and instructed to complete a positive event tracker at least once per day. The positive event tracker asks to describe the event and associated emotions, physical reactions, intensity, duration and individual response. GS2 continues to emphasize increasing exposure and responsiveness to positive events as well as completing a weekly gratitude reflection assignment describing up to 5 things the participant is grateful for. GS3 continues prior activities and adds on acts of kindness for others. During one day in GS3, the participant is requested to perform up to five acts of kindness – all in one day. These acts do not need to be for the same person, and the person(s) may or may not be aware of the act. Once completed, the participant reports on the acts of kindness – what they did and how they felt before, during and after the completed act. GS4 also builds on prior weeks and focuses participants to schedule pleasurable, engaging, and meaningful activities. The participant is encouraged to complete a pleasurable activity alone as well as with others. The participant must also complete an engaging activity in which s/he tends to lose awareness of time or sense of self such as completing a task that challenges skills (e.g., playing a new piece of music; walk or run; engaging in an important yet challenging social interaction or event). And finally, the participant must complete a meaningful activity such as completing a challenging task that one is avoiding, or helping others with a challenging task. The participant logs the pleasurable activities done alone and with others, and also logs the weeks engaging activity and meaningful activity – the emotions and reactions these activities generated and what the individual noted. GS5 continues on building prior progress and introduces creation of positive self-perpetuating interpersonal cycles. In this activity, the participant identifies people within their social network that they would like to further develop or strengthen their relationship with. These could be new relationships (e.g., acquaintances) or existing relationships with friends or family. The participant is then encouraged to use a combination of strategies from previous sessions (increasing responsiveness to positive events, gratitude, and engaging in prosocial acts) to strengthen their relationships. The participant is encouraged to develop a positive activities plan for the future that incorporates activities they liked most, integrating variety, identifying the optimal duration and timing of activities, and involving others for support. Finally, GS6 focuses on relapse prevention. Participants review what they learned in the coaching, how they will maintain and build upon their gains, discuss potential obstacles and establish a plan for addressing them.

**S.6 Representative Lifestyle Analysis from EMAs.**

We only considered EMA, and not smartwatch data for this analysis as some domains, specifically diet and social connection, had no corresponding smartwatch metrics. Specific intervention-relevant EMA targets for this analysis were as below (higher values are better) -

Sleep: percentage of time spent asleep at night compared to phase 1

Exercise: total time (hrs) engaged in mild, moderate, and strenuous exercise compared to phase 1

Diet: reduction in fats and sugars (portions) consumed compared to phase 1

Social Connection: total time (hrs) chatting, in a group, and volunteering compared to phase 1

From the above, each participant had one primary target metric corresponding to their assigned iMAP lifestyle domain. Lifestyle domains not assigned for intervention to the participant are then referred to as their off-target domains. Up to 30 EMAs were obtained during intervention phase 2 corresponding to 6 weeks of intervention with EMA logs made 5 days per week. To facilitate comparison, all depressed mood EMA as well as all domain metrics in each participant were z-score standardized based on their phase 1 EMA data. Off-target domain metrics were averaged together for a combined representation. Any missing EMAs of 30 were replaced with NaNs. Average z score and shaded error bars representing standard error of mean (sem) across participants were plotted for depressed mood, as well as for primary and off target domain changes in intervention phase 2 relative to digital monitoring phase 1.

**S.7 Automated iMAP lifestyle domain assignment.** For this automated ranking, we explored both a simple decision algorithm (DA) and a Google Gemini LLM prompt. The DA followed the same criteria as human assignment. It generated lifestyle domain scores and ranked these based on the top features within a domain (i.e., sleep/exercise/diet/social) in the personalized ML Shapley results, and further considered the independent correlation between feature variables and depression, what ratio of actionable features versus total features within a domain appeared in the top 10 Shapley ranks (actionable features within each domain iMAP are detailed in Supplementary Methods S.5), and whether participants had low satisfaction within that domain indicating need for intervention (see equation 1 below where a higher domain score meant a more suitable iMAP target domain (i.e., sleep/ exercise/ diet/ social connection)) -

Domain Score = $\frac{1}{Domain Rank}*\frac{1}{N_{vars}}\sum\frac{{-\rho}_{var}}{{rank}_{var}}*\% Actionable Vars*\left( \overline{satisfaction} \right)^{-1}$(eq. 1)

This equation has 4 major parts:

1. $\frac{1}{Domain Rank}$ : Here, domain rank is 1-4 based on the order they appear in the top 10 SHAP feature list. Lower numeric value means better result, so the operation is inversed.
2. $\frac{1}{N_{vars}}\sum\frac{{-\rho}_{var}}{{rank}_{var}}$: The summation takes into account the independent correlation between each feature variable and depression and scales it by the relative SHAP rank. Lower ranks should be less weighted hence the division, and large negative correlations are desired (i.e., higher values of the feature variable associated with lower depressed mood), hence the negative weight.
3. $\% Actionable Variables$: ratio of actionable variables within a domain present in the top 10 SHAP feature list and the total number of actionable variables in the domain.
4. $\left( \overline{satisfaction} \right)^{-1}$: We wanted to prioritize domains with low satisfaction so an inverse relationship was used.

The basic domain score equation did not assign any specific weights to the 4 parts above. We also built a further fine-tuned model by iterating the weights of the parts in the above equation based on the target domain preferences made by the coaches across all study participants. This fine-tuned model is shown in equation 2 below. This equation aimed to maximize the domain score by setting all feature correlations in a domain to the max observed correlation (${-\rho}_{max})$ and assigned a weight of 0.9 to this part of the equation. It further added the inverse of lifestyle domain satisfaction to the equation but with a lower weight of 0.1. This fine-tuning was simply done empirically to maximize the percent match in DA-based lifestyle domain assignment with that of the human coach. The domain rank list for the fine-tuned model for all 40 participants is shown in Supplementary Table 1 below.

$Domain Score= \frac{0.9}{N_{vars}}\sum\frac{{-\rho}_{max}}{{rank}_{var}}+0.1*\left( \overline{satisfaction} \right)^{-1}$ (eq.2)

While a decision equation outputs a domain rank list, the additional benefit of an LLM is that it can output logical explanations that may further assist the human coach. However, the LLM will always be a “black box” model even if the prompt provided is transparent and structured. When the naïve decision model aligns with the LLM in the domain ranking order, we harness the LLM's nuanced, context-rich insights while confirming it adheres to the essential decision equation principles. Thus, the prompt given to the Gemini LLM was also based off equation 1 above and the decision making process of the human coach, and is detailed below. Relevant personalized ML model results data was converted into a JSON file and included with the prompt. Notably, maintaining participant privacy and security, the prompt did not include any personal health information (PHI) for any participant –

You are a behavioral health coach that is helping individual patients pick a domain to work on based on their lifestyle data. The data is split into two categories, when the individual experiences high and low depression.

Data Structure:

- 'rank': A numerical identifier for each variable indicating importance. 1 means most important

- 'level_0': Represents the variable name and units.

- 'level_1': Subcategory within each 'level_0', including "MEAN", "COUNT", "STD", and "CI95".

- 'MEAN': Average value.

- 'CORR': Spearmans correlation of variable to depression. This is the same for both low and high groups

- 'STD': Standard deviation.

- 'CI95': 95% confidence interval.

- Relationships:

- Each 'level_0' value has multiple 'level_1' values.

- Each 'level_1' value has corresponding mean, count, std, or CI95 values for the low and high groups.

Domains and relevant variables: The variables belong to one of four possible domains: sleep, exercise, diet, positivity. Ignore all variables that are not listed here.

- Sleep: 'Sleep_percent', 'Sleep_satisfaction'

- Exercise: 'cumm_step_distance', 'cumm_step_speed', 'cumm_step_calorie', 'cumm_step_count', 'heart_rate', 'Exercise_satisfaction', 'exercise_calorie', 'exercise_duration', 'past_day_exercise_moderate', 'past_day_exercise_mild', 'past_day_exercise_strenuous'

- Diet: 'Diet_satisfaction', 'past_day_fats', 'past_day_sugars'

- Positivity: 'Connect_chatpeople', 'Connect_chattime', 'Connect_grouptime', 'Connect_volunteertime', 'Connect_satisfaction', 'Gratitude', 'Reflect_activetime'

IMPORTANT: You must only use the variables specified above when providing justification. Do not include any variables that are not listed.

Your task is to:

Rank the domains from 1-4. This ranking should be weighted based on the ranking of the highest variable, proportion of relevant variables relative to all variables possible,

and strong negative correlation to depression.

Analyze the provided data to rank the intervention domains for behavioral change and suggest specific, actionable recommendations. Recommendations should prioritize positive lifestyle modifications. Rankings for intervention domains for behavioral change should be determined based on number of, rank, and correlation strength of impactful variables that show up.

1. Parameters:

- Do not extrapolate any information that is not given

- If a domain has no relevant variables, it should automatically be ranked last

2. Prioritization:

- Higher ranked variables should be weighted more

- Any domain with variables that have a positive CORR value should incur a penalty

- Variables with stronger negative correlations should be given priority. <-0.4 is considered strong, between [-0.4, -0.2] is considered medium, and between [-0.2 and 0] is considered weak

- Give priority to domains where multiple variables are significant.

3. Suggestions:

- Align with the ranked importance of variables.

- Promote healthy lifestyle changes.

- Ensure recommendations are evidence-based and feasible for implementation.

4. Justification:

- Provide an analysis for each variable based on rank, correlation strength, penalties and how it affects the overall rankings.

Requirements:

- rank the Four Intervention Domains in order by effectiveness of domain: sleep, diet, exercise, or positivity.

- Specific, actionable recommendations that are not drastically different from the current lifestyle.

- Justification referencing data points and variables.

Keep your responses condensed. Provide just the ranking.

**Supplementary Results**

| **Sub** | **1** | **2** | **3** | **4** |  | **Sub** | **1** | **2** | **3** | **4** |
| --- | --- | --- | --- | --- | --- | --- | --- | --- | --- | --- |
| 1 | 0.075 | 0.024 | 0.023 | 0.006 |  | 21 | 0.124 | 0.052 | 0.042 | 0.008 |
| 2 | 0.176 | 0.091 | 0.042 |  |  | 22 | 0.110 | 0.084 | 0.033 | -0.008 |
| 3 | 0.180 | 0.109 | 0.074 | -0.008 |  | 23 | 0.056 | 0.007 | -0.516 |  |
| 4 | 0.651 | 0.122 | 0.056 | 0.032 |  | 24 | 0.086 | 0.064 |  |  |
| 5 | 0.074 | 0.059 | 0.048 |  |  | 25 | 0.220 | 0.118 | 0.095 |  |
| 6 | 0.053 | 0.049 | 0.026 |  |  | 26 | 0.164 | 0.068 | 0.012 |  |
| 7 | 0.077 | 0.074 | 0.050 | 0.041 |  | 27 | 0.126 | 0.039 | 0.028 |  |
| 8 | 0.150 | 0.057 | 0.036 | 0.006 |  | 28 | 0.052 | 0.026 | 0.020 |  |
| 9 | 0.086 | 0.078 | 0.065 |  |  | 29 | 0.131 | 0.122 | 0.088 |  |
| 10 | 0.059 | 0.024 | 0.006 | -0.037 |  | 30 | 0.290 | 0.078 | 0.062 | -0.091 |
| 11 | 0.082 | 0.029 | -0.160 |  |  | 31 | 0.363 | 0.189 | 0.058 | 0.052 |
| 12 | 0.107 | 0.079 | 0.046 |  |  | 32 | 0.208 | 0.108 | 0.046 | 0.035 |
| 13 | 0.186 | 0.130 | 0.039 |  |  | 33 | 0.092 | 0.078 | 0.040 | 0.030 |
| 14 | 0.092 | 0.038 | -0.041 |  |  | 34 | 0.222 | 0.156 | 0.081 | 0.028 |
| 15 | 0.060 | 0.032 | 0.007 | -0.019 |  | 35 | 0.117 | 0.090 | 0.077 |  |
| 16 | 0.134 | 0.073 | 0.054 |  |  | 36 | 0.042 | 0.009 | 0.001 |  |
| 17 | 0.116 | 0.112 | 0.048 | 0.020 |  | 37 | 0.097 |  |  |  |
| 18 | 0.111 | 0.070 | 0.037 |  |  | 38 | 0.107 | 0.081 |  |  |
| 19 | 0.132 | 0.103 | 0.080 |  |  | 39 | 0.109 | 0.104 | -0.011 |  |
| 20 | 0.141 | 0.059 | 0.033 | -0.093 |  | 40 | 0.106 | 0.058 | 0.030 | 0.008 |

**Supplementary Table 1:** Fine-tuned algorithm domain rank list and scores per eq. 2 above for each participant. Domain scores are ordered from highest to lowest. Colored cells denote the iMAP domain assignment made by the human coach that was matched by the decision algorithm in the first or second rank for 38 of 40 participants. Blank cells indicate no score if domain variables were not within the top 10 ranked Shapley variables. Purple: Sleep; Orange: Exercise; Green: Diet; Blue: Social Connection.


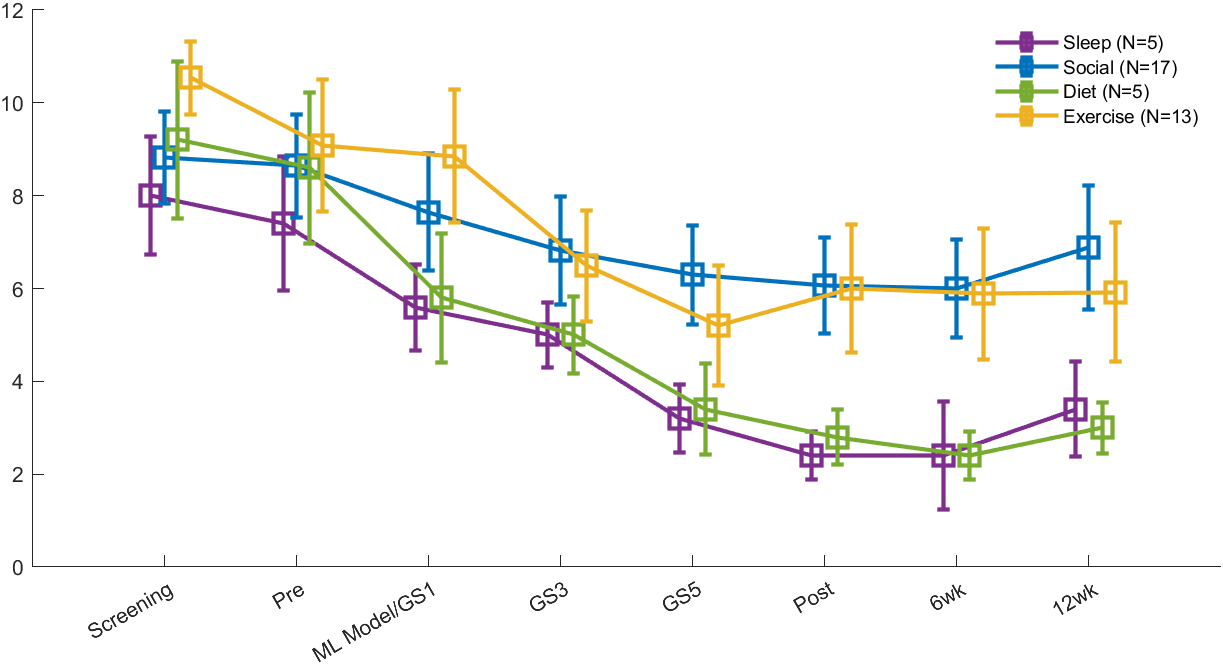


**Supplemental Figure 3.** PHQ9 depression scores during the study period split by the four assigned iMAP domains. Mean $\pm$ standard error (sem) values are shown.

**Supplemental References**

1. Garcı́a-Pérez, M. A. Forced-choice staircases with fixed step sizes: asymptotic and small-sample properties. *Vision Res.* **38**, 1861–1881 (1998).

2. Mishra, J., Anguera, J. A. & Gazzaley, A. Video Games for Neuro-Cognitive Optimization. *Neuron* **90**, 214–218 (2016).

3. Tottenham, N. *et al.* The NimStim set of facial expressions: Judgments from untrained research participants. *Psychiatry Res.* **168**, 242–249 (2009).

4. Greenberg, L. M. & Waldmant, I. D. Developmental Normative Data on The Test of Variables of Attention (T.O.V.A.^TM^). *Journal of Child Psychology and Psychiatry* **34**, 1019–1030 (1993).

5. Wodka, E. L. *et al.* Evidence that response inhibition is a primary deficit in ADHD. *J. Clin. Exp. Neuropsychol.* **29**, 345–356 (2007).

6. Barlow, H. B., Lal, S., Longuet-Higgins, H. C. & Sutherland N.S. The absolute efficiency of perceptual decisions. *Philosophical Transactions of the Royal Society of London. B, Biological Sciences* **290**, 71–82 (1980).

7. Vandierendonck, A. A comparison of methods to combine speed and accuracy measures of performance: A rejoinder on the binning procedure. *Behav. Res. Methods* **49**, 653–673 (2017).

8. Heeger, D. & Landy, M. Signal Detection Theory. *Encyclopedia of perception* 887–892 (2009).

9. Eriksen, B. A. & Eriksen, C. W. Effects of noise letters upon the identification of a target letter in a nonsearch task. *Percept. Psychophys.* **16**, 143–149 (1974).

10. Lavie, N., Hirst, A., de Fockert, J. W. & Viding, E. Load Theory of Selective Attention and Cognitive Control. *J. Exp. Psychol. Gen.* **133**, 339–354 (2004).

11. Shipstead, Z., Harrison, T. L. & Engle, R. W. Working Memory Capacity and Visual Attention: Top-Down and Bottom-Up Guidance. *Quarterly Journal of Experimental Psychology* **65**, 401–407 (2012).

12. Sternberg, S. High-Speed Scanning in Human Memory. *Science (1979).* **153**, 652–654 (1966).

13. López-Martín, S., Albert, J., Fernández-Jaén, A. & Carretié, L. Emotional response inhibition in children with attention-deficit/hyperactivity disorder: neural and behavioural data. *Psychol. Med* **45**, (2015).

14. López-Martín, S., Albert, J., Fernández-Jaén, A. & Carretié, L. Emotional distraction in boys with ADHD: Neural and behavioral correlates. *Brain Cogn.* **83**, (2013).

15. Shah, R. V. *et al.* Personalized machine learning of depressed mood using wearables. *Transl. Psychiatry* **11**, 338 (2021).

16. Nan, J. *et al.* Personalized Machine Learning-Based Prediction of Wellbeing and Empathy in Healthcare Professionals. *Sensors* **24**, 2640 (2024).

17. Gemming, L., Utter, J. & Ni Mhurchu, C. Image-Assisted Dietary Assessment: A Systematic Review of the Evidence. *J. Acad. Nutr. Diet.* **115**, 64–77 (2015).

18. Stumbo, P. J. New technology in dietary assessment: a review of digital methods in improving food record accuracy. *Proceedings of the Nutrition Society* **72**, 70–76 (2013).

19. Francis, H. M. *et al.* A brief diet intervention can reduce symptoms of depression in young adults – A randomised controlled trial. *PLoS One* **14**, e0222768 (2019).

20. Opie, R. S., O’Neil, A., Itsiopoulos, C. & Jacka, F. N. The impact of whole-of-diet interventions on depression and anxiety: a systematic review of randomised controlled trials. *Public Health Nutr.* **18**, 2074–2093 (2015).

21. Opie, R. S., O’Neil, A., Jacka, F. N., Pizzinga, J. & Itsiopoulos, C. A modified Mediterranean dietary intervention for adults with major depression: Dietary protocol and feasibility data from the SMILES trial. *Nutr. Neurosci.* **21**, 487–501 (2018).

22. Parletta, N. *et al.* A Mediterranean-style dietary intervention supplemented with fish oil improves diet quality and mental health in people with depression: A randomized controlled trial (HELFIMED). *Nutr. Neurosci.* **22**, 474–487 (2019).

23. Francis, H. & Stevenson, R. Validity and test-retest reliability of a short dietary questionnaire to assess intake of saturated fat and free sugars: a preliminary study. *Journal of Human Nutrition and Dietetics* **26**, 234–242 (2013).

24. Jaiswal, S. *et al.* Breath-Focused Mindfulness and Compassion Training in Parent-Child Dyads: Pilot Intervention Study. *JMIR Form. Res.* **9**, e69607–e69607 (2025).

25. Jaiswal, S. *et al.* Design and Implementation of a Brief Digital Mindfulness and Compassion Training App for Health Care Professionals: Cluster Randomized Controlled Trial. *JMIR Ment. Health* **11**, e49467 (2024).

26. Schuman-Olivier, Z. *et al.* Mindfulness and Behavior Change. *Harv. Rev. Psychiatry* **28**, 371–394 (2020).

27. Werner, K. & Gross, J. J. Emotion Regulation and Psychopathology:  A Conceptual Framework. in *Emotion regulation and psychopathology: A transdiagnostic approach to etiology and treatment.* (eds. Kring, A. M. & Sloan, D. M.) 13–37 ( THE GUILFORD PRESS, 2010).

28. Gawande, R. *et al.* Mindfulness Training Enhances Self-Regulation and Facilitates Health Behavior Change for Primary Care Patients: a Randomized Controlled Trial. *J. Gen. Intern. Med.* **34**, 293–302 (2019).

29. Britton, W. *et al.* Meta-analytic review of self-regulation processes in mindfulness-based cognitive therapy and mindfulness-based stress reduction. *Prospero* (2016).

30. Brewer, J. A., Elwafi, H. M. & Davis, J. H. Craving to quit: Psychological models and neurobiological mechanisms of mindfulness training as treatment for addictions. *Psychology of Addictive Behaviors* **27**, 366–379 (2013).

31. Fulwiler, C., Brewer, J. A., Sinnott, S. & Loucks, E. B. Mindfulness-Based Interventions for Weight Loss and CVD Risk Management. *Curr. Cardiovasc. Risk Rep.* **9**, 46 (2015).

32. Loucks, E. B. *et al.* Mindfulness and Cardiovascular Disease Risk: State of the Evidence, Plausible Mechanisms, and Theoretical Framework. *Curr. Cardiol. Rep.* **17**, 112 (2015).

33. Levinson, D. B., Stoll, E. L., Kindy, S. D., Merry, H. L. & Davidson, R. J. A mind you can count on: validating breath counting as a behavioral measure of mindfulness. *Front. Psychol.* **5**, (2014).

34. Mishra, J. *et al.* Closed-loop digital meditation for neurocognitive and behavioral development in adolescents with childhood neglect. *Transl. Psychiatry* **10**, 153 (2020).

35. Ziegler, D. A. *et al.* Closed-loop digital meditation improves sustained attention in young adults. *Nat. Hum. Behav.* **3**, 746–757 (2019).

36. Taylor, C. T., Lyubomirsky, S. & Stein, M. B. Upregulating the positive affect system in anxiety and depression: Outcomes of a positive activity intervention. *Depress. Anxiety* **34**, 267–280 (2017).

37. Ramanathan, D. *et al.* Modulation of Posterior Default Mode Network Activity During Interoceptive Attention and Relation to Mindfulness. *Biological Psychiatry Global Open Science* **4**, 100384 (2024).

38. van Buuren, S. & Groothuis-Oudshoorn, K. mice: Multivariate imputation by chained equations in R. *J. Stat. Softw.* **45**, 1–67 (2011).

39. Cowley, B. R. *et al.* DataHigh: graphical user interface for visualizing and interacting with high-dimensional neural activity. *J. Neural Eng.* **10**, 66012 (2013).

40. Brownless, J. Nested Cross-Validation for Machine Learning with Python. https://machinelearningmastery.com/nested-cross-validation-for-machine-learning-with-python/ (2020).

41. Shah, R. V. *et al.* Personalized machine learning of depressed mood using wearables. *Transl. Psychiatry* **11**, 338 (2021).

42. Nan, J. *et al.* Personalized Machine Learning-Based Prediction of Wellbeing and Empathy in Healthcare Professionals. *Sensors* vol. 24 Preprint at https://doi.org/10.3390/s24082640 (2024).

43. Nan, J., Grennan, G., Ravichandran, S., Ramanathan, D. & Mishra, J. Neural activity during inhibitory control predicts suicidal ideation with machine learning. *NPP—Digital Psychiatry and Neuroscience* **2**, 10 (2024).

44. Asarnow, L. D. & Manber, R. Cognitive Behavioral Therapy for Insomnia in Depression. *Sleep Med. Clin.* **14**, 177–184 (2019).

45. Hertenstein, E. *et al.* Cognitive behavioral therapy for insomnia in patients with mental disorders and comorbid insomnia: A systematic review and meta-analysis. *Sleep Med. Rev.* **62**, (2022).

46. Noetel, M. *et al.* Effect of exercise for depression: systematic review and network meta-analysis of randomised controlled trials. *BMJ* **384**, (2024).

47. Sallis, R. Exercise is medicine: a call to action for physicians to assess and prescribe exercise. *Phys. Sportsmed.* **43**, 22–26 (2015).

48. Opie, R. S., O’Neil, A., Jacka, F. N., Pizzinga, J. & Itsiopoulos, C. A modified Mediterranean dietary intervention for adults with major depression: Dietary protocol and feasibility data from the SMILES trial. *Nutr. Neurosci.* **21**, 487–501 (2018).

49. Opie, R. S., O’Neil, A., Itsiopoulos, C. & Jacka, F. N. The impact of whole-of-diet interventions on depression and anxiety: A systematic review of randomised controlled trials. *Public Health Nutrition* vol. 18 2074–2093 Preprint at https://doi.org/10.1017/S1368980014002614 (2015).

50. Taylor, C. T., Lyubomirsky, S. & Stein, M. B. Upregulating the positive affect system in anxiety and depression: Outcomes of a positive activity intervention. *Depress. Anxiety* **34**, 267–280 (2017).

51. Taylor, C. T. *et al.* Amplification of Positivity Treatment for Anxiety and Depression: A Randomized Experimental Therapeutics Trial Targeting Social Reward Sensitivity to Enhance Social Connectedness. *Biol. Psychiatry* **0**, (2023).

**CONSORT 2025 checklist of information to include when reporting a randomised trial***

| **Section / Topic** | **No** | **CONSORT 2025 checklist item description** | **Reported on page no.** |
| --- | --- | --- | --- |
| **Title and abstract** | | |  |
| Title and structured abstract | 1a | Identification as a randomised trial | 2 |
|  | 1b | Structured summary of the trial design, methods, results, and conclusions | 2 |
| **Open science** | | |  |
| Trial registration | 2 | Name of trial registry, identifying number (with URL) and date of registration | 4 |
| Protocol and statistical analysis plan | 3 | Where the trial protocol and statistical analysis plan can be accessed | 4 |
| Data sharing | 4 | Where and how the individual de-identified participant data (including data dictionary), statistical code and any other materials can be accessed | N/A |
| Funding and conflicts of interest | 5a | Sources of funding and other support (e.g., supply of drugs), and role of funders in the design, conduct, analysis and reporting of the trial | 22 |
|  | 5b | Financial and other conflicts of interest of the manuscript authors | 21 |
| **Introduction** | | |  |
| Background and rationale | 6 | Scientific background and rationale | 3-4 |
| Objectives | 7 | Specific objectives related to benefits and harms | 3-4 |
| **Methods** | | |  |
| Patient and public involvement | 8 | Details of patient or public involvement in the design, conduct and reporting of the trial | 4 |
| Trial design | 9 | Description of trial design including type of trial (e.g., parallel group, crossover), allocation ratio, and framework (e.g., superiority, equivalence, non-inferiority, exploratory) | 4-5 |
| Changes to trial protocol | 10 | Important changes to the trial after it commenced including any outcomes or analyses that were not prespecified, with reason | N/A |
| Trial setting | 11 | Settings (e.g., community, hospital) and locations (e.g., countries, sites) where the trial was conducted | 4-5 |
| Eligibility criteria | 12a | Eligibility criteria for participants | 4 |
|  | 12b | If applicable, eligibility criteria for sites and for individuals delivering the interventions (e.g., surgeons, physiotherapists) | 5 |
| Intervention and comparator | 13 | Intervention and comparator with sufficient details to allow replication. If relevant, where additional materials describing the intervention and comparator (e.g., intervention manual) can be accessed | 5-10 |
| Outcomes | 14 | Pre-specified primary and secondary outcomes, including the specific measurement variable (e.g., systolic blood pressure), analysis metric (e.g., change from baseline, final value, time to event), method of aggregation (e.g., median, proportion), and time point for each outcome | 8-9 |
| Harms | 15 | How harms were defined and assessed (e.g., systematically, non-systematically) | 6-7 |
| Sample size | 16a | How sample size was determined, including all assumptions supporting the sample size calculation | 4 |
|  | 16b | Explanation of any interim analyses and stopping guidelines |  |
| Randomisation: |  |  | N/A |
| Sequence generation | 17a | Who generated the random allocation sequence and the method used | N/A |
|  | 17b | Type of randomisation and details of any restriction (e.g., stratification, blocking and block size) | 4 |
| Allocation concealment mechanism | 18 | Mechanism used to implement the random allocation sequence (e.g., central computer/telephone; sequentially numbered, opaque, sealed containers), describing any steps to conceal the sequence until interventions were assigned | N/A |
| Implementation | 19 | Whether the personnel who enrolled and those who assigned participants to the interventions had access to the random allocation sequence | N/A |
| Blinding | 20a | Who was blinded after assignment to interventions (e.g., participants, care providers, outcome assessors, data analysts) | N/A |
|  | 20b | If blinded, how blinding was achieved and description of the similarity of interventions | N/A |
| Statistical methods | 21a | Statistical methods used to compare groups for primary and secondary outcomes, including harms | 4-10 |
|  | 21b | Definition of who is included in each analysis (e.g., all randomised participants), and in which group | 4-10 |
|  | 21c | How missing data were handled in the analysis | 8-10 |
|  | 21d | Methods for any additional analyses (e.g., subgroup and sensitivity analyses), distinguishing prespecified from post-hoc | 8-10 |
| **Results** | | |  |
| Participant flow, including flow diagram | 22a | For each group, the numbers of participants who were randomly assigned, received intended intervention, and were analysed for the primary outcome | 11 |
|  | 22b | For each group, losses and exclusions after randomisation, together with reasons | 11 |
| Recruitment | 23a | Dates defining the periods of recruitment and follow-up for outcomes of benefits and harms | N/A |
|  | 23b | If relevant, why the trial ended or was stopped | N/A |
| Intervention and comparator delivery | 24a | Intervention and comparator as they were actually administered (e.g., where appropriate, who delivered the intervention/comparator, how participants adhered, whether they were delivered as intended [fidelity]) | 5 |
|  | 24b | Concomitant care received during the trial for each group | N/A |
| Baseline data | 25 | A table showing baseline demographic and clinical characteristics for each group | 11 |
| Numbers analysed,  outcomes and estimation | 26 | For each primary and secondary outcome, by group:   - the number of participants included in the analysis - the number of participants with available data at the outcome time point - result for each group, and the estimated effect size and its precision (such as 95% confidence interval) - for binary outcomes, presentation of both absolute and relative effect size | 14-18 |
| Harms | 27 | All harms or unintended events in each group | N/A |
| Ancillary analyses | 28 | Any other analyses performed, including subgroup and sensitivity analyses, distinguishing pre-specified from post-hoc | N/A |
| **Discussion** | | |  |
| Interpretation | 29 | Interpretation consistent with results, balancing benefits and harms, and considering other relevant evidence | 18-21 |
| Limitations | 30 | Trial limitations, addressing sources of potential bias, imprecision, generalisability, and, if relevant, multiplicity of analyses | 21 |

*We strongly recommend reading this statement in conjunction with the CONSORT 2025 Explanation and Elaboration and/or the CONSORT 2025 Expanded Checklist for important clarifications on all the items. We also recommend reading relevant CONSORT extensions. See [www.consort-spirit.org](http://www.consort-spirit.org).

Citation: Hopewell S, Chan AW, Collins GS, Hróbjartsson A, Moher D, Schulz KF, et al. CONSORT 2025 Statement: updated guideline for reporting randomised trials. BMJ. 2025; 388:e081123. <https://dx.doi.org/10.1136/bmj-2024-081123>.

© 2025 Hopewell et al. This is an Open Access article distributed under the terms of the Creative Commons Attribution License (<https://creativecommons.org/licenses/by/4.0/>), which permits unrestricted use, distribution, and reproduction in any medium, provided the original work is properly cited

**CONSORT Flow Diagram for the Personalized Mood Augmentation trial**

Analyzed for primary outcome Intent-to-treat (n=50)

Analyzed for primary outcome per protocol (n=40)

Excluded from analysis (n=0)

Follow-Up

Analysis

Completed intervention (n=40)

Discontinued intervention (time constraint) (n=10)

Lost to follow-up for primary outcome (n= 0)

Allocation

Allocated to intervention (n= 50)

Excluded (n= 52)

Not meeting inclusion criteria (n=22)

Declined to participate (n= 30)

Other reasons (n= 0)

Enrolment

Assessed for eligibility (n= 102)
